# Fast Decomposition of Three-Component Spectra of Fluorescence Quenching by White and Grey Methods of Data Modeling

**DOI:** 10.1007/s10895-018-2224-5

**Published:** 2018-04-03

**Authors:** Andrzej J. Kałka, Andrzej M. Turek

**Affiliations:** Faculty of Chemistry, Jagiellonian University, 2 Gronostajowa St, 30 387 Cracow, Poland

**Keywords:** Multiple curve resolution, Stern-Volmer plot, Difference fluorescence spectra, Rank annihilation factor analysis, Non-linear least squares optimization

## Abstract

‘White’ and ‘grey’ methods of data modeling have been employed to resolve the heterogeneous fluorescence from a fluorophore mixture of 9-cyanoanthracene (CNA), 10-chloro-9-cyanoanthracene (ClCNA) and 9,10-dicyanoanthracene (DCNA) into component individual fluorescence spectra. The three-component spectra of fluorescence quenching in methanol were recorded for increasing amounts of lithium bromide used as a quencher. The associated intensity decay profiles of differentially quenched fluorescence of single components were modeled on the basis of a linear Stern-Volmer plot. These profiles are necessary to initiate the fitting procedure in both ‘white’ and ‘grey’ modeling of the original data matrices. ‘White’ methods of data modeling, called also ‘hard’ methods, are based on chemical/physical laws expressed in terms of some well-known or generally accepted mathematical equations. The parameters of these models are not known and they are estimated by least squares curve fitting. ‘Grey’ approaches to data modeling, also known as hard-soft modeling techniques, make use of both hard-model and soft-model parts. In practice, the difference between ‘white’ and ‘grey’ methods lies in the way in which the ‘crude’ fluorescence intensity decays of the mixture components are estimated. In the former case they are given in a functional form while in the latter as digitized curves which, in general, can only be obtained by using dedicated techniques of factor analysis. In the paper, the initial values of the Stern-Volmer constants of pure components were evaluated by both ‘point-by-point’ and ‘matrix’ versions of the method making use of the concept of wavelength dependent intensity fractions as well as by the rank annihilation factor analysis applied to the data matrices of the difference fluorescence spectra constructed in two ways: from the spectra recorded for a few excitation lines at the same concentration of a fluorescence quencher or classically from a series of the spectra measured for one selected excitation line but for increasing concentration of the quencher. The results of multiple curve resolution obtained by all types of the applied methods have been scrutinized and compared. In addition, the effect of inadequacy of sample preparation and increasing instrumental noise on the shape of the resolved spectral profiles has been studied on several datasets mimicking the measured data matrices.

**Graphical Abstract:**
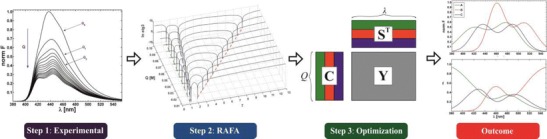
ᅟ

ᅟ

## Introduction

The rapidly developing methods of chemical analysis are nowadays those involving self-modeling curve resolution (SMCR) of a spectral data matrix representing a multi-component mixture of spectrally active components. The main objective of such approaches is to decompose the measured data matrix into the product of two matrices: first containing the spectra of pure components and another one representing their relative concentrations. Preliminary step in this analysis consists, however, of decomposition of the original data matrix into the product of the matrices containing the so called abstract spectral and concentration profiles. Typically, this is achieved by using the Jacobi algorithm of the principal component analysis (PCA) or its more elegant version called the singular value decomposition (SVD) [1]. Upon the use of a proper transformation matrix the abstract matrices could easily be converted into the predicted profiles of both types of variability [2].

For the first time, the concept of SMCR was successfully elaborated and applied in the early 1970s by Lawton and Sylvestre [3]. The analyzed data matrix was a spectrophotometric dataset representing a mixture of only two chemical species. Since the proposed method was based on two rather obvious premises concerning non-negativity of the predicted spectra of pure components as well as non-negativity of the coefficients of a linear combination used to build up each measured two-component spectrum, the obtained solutions were not unique and classified later on as belonging to the category of soft data modeling. Soon, an attempt to extend this approach to a three-component system was made by Ohta [4]. By keeping the same minimum set of constraints and imposing a constant value on all three elements of one vector of the transformation matrix, the three-dimensional problem was reduced to two dimensions. This allowed to determine an appropriate set of the elements of the remaining two other vectors of the transformation matrix and consequently also to visualize the area of feasible solutions (AFS) for the pure component spectra. The selection of this so called T-space representation of the three-component data was carried out by the Monte Carlo method producing feasible spectral bands for all components of the three-component system [4]. Almost 30 years later this approach was effectively improved by Leger and Wentzell and introduced to the literature as the dynamic Monte Carlo SMCR [5]. In the meantime, the random AFS generation for three component systems was neatly replaced by an approach taking advantage of the ideas developed by computational geometricians. This was commenced by Borgen and Kovalski who developed the mathematical tools for confining the T-space convex hulls related to AFS [6]. The so called Borgen plots, preserving the two intrinsic assumptions of soft data modeling, were then successively modified by adding some other constraints narrowing the bands of the AFS computed spectra and concentration profiles [7–11] .

The classical soft modeling methods mentioned above [3–11] provide possibly the best estimated pure component spectra but sometimes only the selection of the purest measured spectra is required and made. Such spectra can easily be sought by using the criterion of maximal spectral dissimilarity as demonstrated by Cruciani et al. [12] or by applying any other non-factor analysis method employing this concept such as simple-to-use-interactive-self-modeling-mixture-analysis (SIMPLISMA) [13], orthogonal projection approach (OPA) [14] or alternating least squares (ALS) [15]. The same goal is also achieved using iterative target transformation factor analysis (ITTFA) [16, 17]. Some other less common rational curve resolution methodologies are briefly characterized in review papers by Jiang and Ozaki [18] or Jiang et al. [19].

The regions of existence of unique contributions from single components in some portions of the measured data matrix (selective regions) as well as those signalizing the absence of a contribution from a specific component (“zero’ regions) are of uttermost importance for reducing the number of feasible solutions and reliability of the resolved profiles. These regions were intensively utilized in multivariate curve resolution of overlapping chromatographic peaks in HPLC-DAD chromatograms [20–23]. A simple tutorial on how to use this information obtained from evolutionary rank analysis of the data matrix provided by Maeder’s evolving factor analysis (EFA) [20, 21] and Kvalheim and Liang heuristic evolving latent projections (HELP) [22] has been reliably crafted by Toft [23].

A significant improvement or even unique curve resolution can be achieved if instead of one data matrix two or more matrices with altered evolution of the concentration profiles are factor-analyzed. These model-free techniques include generalized rank annihilation method (GRAM) [24, 25] and/or Kubista’s approach [26] for a pair of two-way matrices as well as parallel factor analysis (PARAFAC) [27–29] for a three-way data array (a stack of matrices). In this context, an instructive example of effective application of such trilinear decomposition technique to several excitation-emission matrices (EEMs) measured for different concentrations of a fluorescence quenching agent has been provided by Wentzell et al. [30]. As highlighted by these authors, inevitable Rayleigh and Raman scattering caused by the solvent molecules and possible primary absorption of the quencher lead, however, to apparently distorted EEMs which hardly can be corrected with no left traces.

In the case of a single experimental data matrix the same goal can be accomplished quite often by hard modelling that is by taking into account the existing physical/chemical laws responsible for evolution of each individual concentration profile. The evolving concentration profile can be directly expressed as a function of time, pH or another non-random variable using the relevant mathematical formula (white method) or represented by its digitized form obtained by a partial usage of the information concerning the existing law combined with a complementary application of some soft-model approach [31–34]. The latter method is called a grey method.

In this paper a detailed analysis and comparison of the results obtained using white (hard) and grey (hard+soft) MCR methodologies applied to resolve the spectra of a three-component system of quenched fluorescence has been included. The presentation goes as follows: in Second section with five subsections the essential theoretical foundations of the employed methods are explicitly stated. Third section gives details of experimental conditions and sample preparation. Fourth section provides a discussion of the obtained results and is organized around two subsections. In the first subsection the results obtained for simulated dataset are examined while the second subsection dwells on the analysis of the results referring to real experimental dataset. In closing Fifth section the outcome of this study is succinctly summarized in four subsections.

## Theoretical Background

### Fluorescence Quenching

It is well known that in the case of collisional fluorescence quenching the ratio of the integrated intensity of the fluorescence spectra in the absence and in the presence of a specified amount of quencher, *Q*, can be replaced by the ratio of the observed signal intensities at any emission wavelength, ${F}_0^{\lambda }/{F}^{\lambda }$, if the shape of the emission spectrum is not modified by quenching. If so, then the ratio of fluorescence intensities, ${F}_0^{\lambda }/{F}^{\lambda }$, increases linearly with the quencher concentration. This dependence shown below is called the linear Stern-Volmer equation

1 $$ \frac{F_0^{\lambda }}{F^{\lambda }}=1+{K}_{\mathrm{SV}}Q $$

where *K*_SV_ = *k*_*q*_*τ*_0_ and *k*_*q*_ is the quenching rate constant, *τ*_0_ is the singlet state lifetime in the absence of quencher, and *λ* designates the selected emission wavelength. Deviations to the simple Stern-Volmer plots defined above can be numerous as discussed in [35], however, at sufficiently low concentrations of the quencher (usually below 0.1 M) this linear relationship holds true for all components of the fluorophore mixture.

**Fig. 1:**
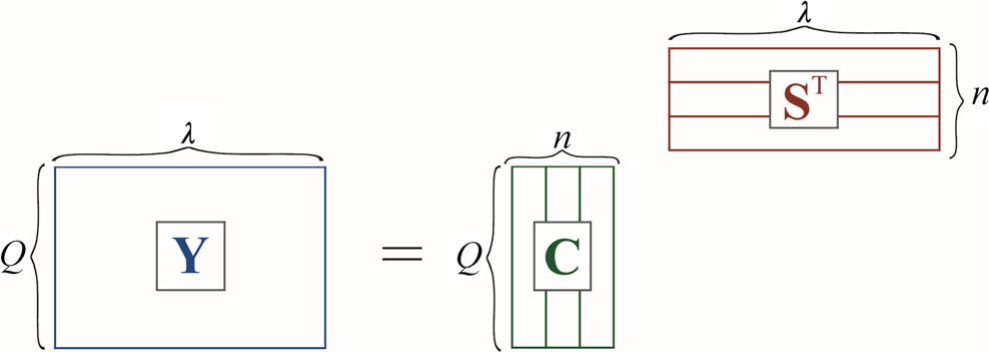
Decomposition of multi-component data matrix of fluorescence quenching, **Y**, into a product of two matrices **C** and **S**^**T**^ containing resolved intensity decays and spectra of pure fluorophores, respectively

In general, an original data matrix **Y** containing, in its rows, the multi-component spectra of the quenched fluorescence recorded for successive quenching experiments can be represented by a product of two matrices: the first with decays of the emission intensities of individual components caused by quenching, **C**, and the second (transposed) matrix, **S**^**T**^**,** having in its rows the fluorescence spectra of those pure components. The dimension of these matrices are defined by the following numbers: *n* – number of chemical components, *Q* – number of added portions of quencher, and *λ* - number of the used emission wavelengths (see Fig. 1). Hence, the matrix **C** is a matrix equivalent to the matrix of individual pure concentration profiles resolved from overlapping chromatographic structures [20–24].

All the above mentioned matrices appear in the following equation

2 $$ \mathbf{Y}={\mathbf{CS}}^{\mathbf{T}} $$

If matrix **C** is known then upon simple transformation the spectral profiles of all fluorescent components are given by

3 $$ {\mathbf{S}}^{\mathbf{T}}={\left({\mathbf{C}}^{\mathbf{T}}\mathbf{C}\right)}^{-1}{\mathbf{C}}^{\mathbf{T}}\mathbf{Y}={\mathbf{C}}^{+}\mathbf{Y} $$

Matrix **C**^**+**^ in Eq. (3) is called the left pseudoinverse of matrix **C**. Thus, the main task, as regards the decomposition of the spectra of multi-component mixture of fluorophores, consists in finding the Stern-Volmer constants, *K*_SV_, for all involved components.

### Rank Annihilation Factor Analysis

#### *τ*‐*RAFA*

Recently, it has been demonstrated that the successful estimation of *K*_SV_^'^*s* for a three-component mixture of fluorophores can be easily carried out [36] by using an iterative version of rank annihilation factor analysis (RAFA) as proposed by Davidson et al. [37, 38]. In order to apply this method it is necessary to measure a few series of quenched fluorescence spectra with various excitation lines. Then the data matrices are constructed in such a way that each matrix, **M**_*Q*_, contains in its rows the spectra recorded successively for all selected excitation lines but for a specified amount of the quencher. Naturally, a reference matrix, **M**_0_, for unquenched fluorescence is generated alike. A three-component fluorescence spectrum measured with a specified excitation wavelength, *λ*, is thus a sum of the spectra of particular components referring to the same excitation wavelength, as given below

4 $$ {\mathbf{m}}^{\lambda }={\mathbf{m}}_Q^{\mathrm{A}}+{\mathbf{m}}_Q^{\mathrm{B}}+{\mathbf{m}}_Q^{\mathrm{C}} $$

On the right side of Eq. (4) some expected *λ* symbols are omitted for simplicity, e.g. it should be ${\mathbf{m}}_Q^{\lambda, \mathrm{A}}$ but is ${\mathbf{m}}_Q^{\mathrm{A}}$, and so on. The next step involves construction of a difference matrix, **D**_*Q*_

5 $$ {\mathbf{D}}_Q={\mathbf{M}}_0-\tau {\mathbf{M}}_Q $$

with successive *λ*-dependent rows defined as follows

6 $$ {\mathbf{d}}_Q^{\lambda }=\left({\mathbf{m}}_0^{\mathrm{A}}-\tau {\mathbf{m}}_Q^{\mathrm{A}}\right)+\left({\mathbf{m}}_0^{\mathrm{B}}-\tau {\mathbf{m}}_Q^{\mathrm{B}}\right)+\left({\mathbf{m}}_0^{\mathrm{C}}-\tau {\mathbf{m}}_Q^{\mathrm{C}}\right) $$

where *τ* is a floating parameter. By stepping *τ* in its predefined range it is possible to find such a value of *τ* that the Stern-Volmer dependence for one of the components, say component A, can be satisfied

7 $$ {\mathbf{m}}_0^{\mathrm{A}}=\left(1+{K}_{\mathrm{SV}}^{\mathrm{A}}Q\right){\mathbf{m}}_Q^{\mathrm{A}}=\tau {\mathbf{m}}_Q^{\mathrm{A}} $$

**Fig. 2:**
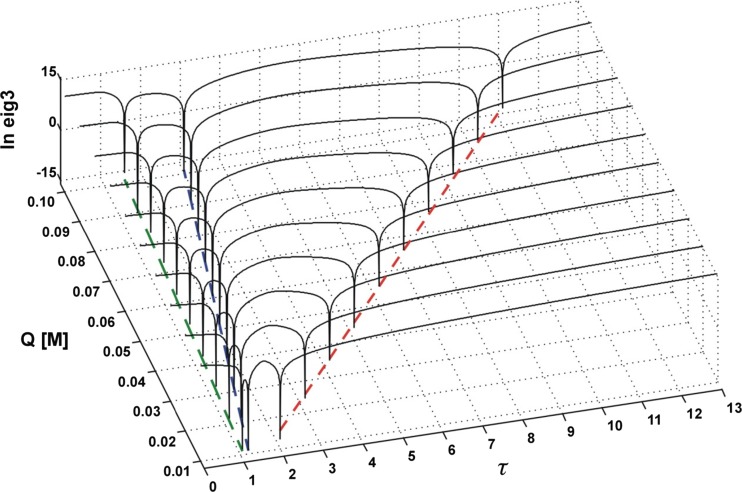
*τ*-RAFA analysis of the covariance matrix of an ideal three-component system of quenched fluorescence

The spectral contribution from component A to the overall fluorescence intensity is then lost

8 $$ {\mathbf{d}}_Q^{\lambda }=0+\left({\mathbf{m}}_0^{\mathrm{B}}-\tau {\mathbf{m}}_Q^{\mathrm{B}}\right)+\left({\mathbf{m}}_0^{\mathrm{C}}-\tau {\mathbf{m}}_Q^{\mathrm{C}}\right) $$

which is reflected in a substantial cutdown of the third eigenvalue of the covariance matrix ${\mathbf{D}}_Q{\mathbf{D}}_Q^{\mathbf{T}}$ due to efficient reduction of the number of significant components of the spectral mixture from three to two. The optimum value of the floating parameter, *τ*, is found for each quencher concentration, *Q*, by tracing the changes in the third eigenvalue of the covariance matrix ${\mathbf{D}}_Q{\mathbf{D}}_Q^{\mathbf{T}}$ as a function of *τ*. Then a plot is made which in the case of component A gives

9 $$ \tau (Q)=1+{K}_{\mathrm{SV}}^{\mathrm{A}}Q $$

with the slope equal to the Stern-Volmer constant of component A. The whole procedure is illustrated in Fig. 2 shown below.

#### *κ*‐*RAFA*

A much more simple alternative to the *τ*-RAFA approach described above is also conceivable. The rows of a difference matrix **D** can be formed not for different excitation lines and constant quencher concentration but conversely for different quencher concentrations and one excitation line as demonstrated below for a specified quencher concentration, *Q*,

10 $$ {\mathbf{d}}_Q={\mathbf{m}}_0^{\mathrm{A}}-\left(1+\kappa Q\right){\mathbf{m}}_Q^{\mathrm{A}}+{\mathbf{m}}_0^{\mathrm{B}}-\left(1+\kappa Q\right){\mathbf{m}}_Q^{\mathrm{B}}+{\mathbf{m}}_0^{\mathrm{C}}-\left(1+\kappa Q\right){\mathbf{m}}_Q^{\mathrm{C}} $$

The iterative parameter, marked here as *κ*, becomes equivalent to Stern-Volmer constant. In other words, in the case of a three-component system, the expected value of a quenching constant should be equal to the *κ* value corresponding to the minimum value of the third eigenvalue of the covariance matrix formed from the difference matrix **D**.

In Authors’ opinion this simple *κ*-RAFA approach should be called a ‘direct’ method while the word ‘indirect’ would rather be reserved for the *τ*-RAFA methodology. In the present article the performance of both methods as well as the effect of the type of noise and its magnitude on the final results have been carefully investigated (for details see Results and Discussion).

### ‘Point-by-Point’ Optimization of Stern-Volmer Constants

Having at hand some initial estimates for the Stern-Volmer constants it is possible to refine these values and then to initiate the process of resolution of the multi-component fluorescence spectra. Historically, the first approach to this problem was made by Sherwin Lehrer [39]. Originally applied to determine a fraction of unquenched fluorescence it was based on the ‘point’ Stern-Volmer dependence as defined by Eq. (1). (For better readability of equations, in the next portions of this article the symbols *λ* and SV will be omitted). The above cited author introduced a concept of the fraction of the emission intensity of the *i*-th component, *f*_*i*_, defined as the ratio of the contribution of its fluorescence intensity to the overall fluorescence intensity of the **unquenched** emission, at a fixed emission wavelength. For a three-component system, with components A, B and C, it reads like below

11 $$ {f}_{\mathrm{A}}=\frac{F_0^{\mathrm{A}}}{F_0}=\frac{F_0^{\mathrm{A}}}{F_0^{\mathrm{A}}+{F}_0^{\mathrm{B}}+{F}_0^{\mathrm{C}}} $$

By modifying the classical expression for the Stern-Volmer dependence through introduction of the difference between the ‘point’ fluorescence intensities of the unquenched emission, *F*_0_, and the quenched emission, *F*, one gets

12 $$ \frac{F_0-F}{F_0}=\frac{\Delta F}{F_0}=\frac{f_{\mathrm{A}}{K}_{\mathrm{A}}Q}{1+{K}_{\mathrm{A}}Q}+\frac{f_{\mathrm{B}}{K}_{\mathrm{B}}Q}{1+{K}_{\mathrm{B}}Q}+\frac{f_{\mathrm{C}}{K}_{\mathrm{C}}Q}{1+{K}_{\mathrm{C}}Q} $$

Upon bringing the above expression to the common denominator, a third degree rational function of *Q* is obtained

13 $$ g(Q)=\frac{\Delta F}{F_0}=\frac{a_0+{a}_1Q+{a}_2{Q}^2+{a}_3{Q}^3}{b_0+{b}_1Q+{b}_2{Q}^2+{b}_3{Q}^3}, $$

with the related parameters *a* and *b* calculated as shown below

14 $$ {\displaystyle \begin{array}{l}{a}_0=0\\ {}{a}_1={f}_{\mathrm{A}}{K}_{\mathrm{A}}+{f}_{\mathrm{B}}{K}_{\mathrm{B}}+{f}_{\mathrm{C}}{K}_{\mathrm{C}}\\ {}{a}_2={f}_{\mathrm{A}}{K}_{\mathrm{A}}\left({K}_{\mathrm{B}}+{K}_{\mathrm{C}}\right)+{f}_{\mathrm{B}}{K}_{\mathrm{B}}\left({K}_{\mathrm{A}}+{K}_{\mathrm{C}}\right)+{f}_{\mathrm{C}}{K}_{\mathrm{C}}\left({K}_{\mathrm{A}}+{K}_{\mathrm{B}}\right)\\ {}{a}_3={K}_{\mathrm{A}}{K}_{\mathrm{B}}{K}_{\mathrm{C}}\left({f}_{\mathrm{A}}+{f}_{\mathrm{B}}+{f}_{\mathrm{C}}\right)\\ {}{b}_0=1\\ {}{b}_1={K}_{\mathrm{A}}+{K}_{\mathrm{B}}+{K}_{\mathrm{C}}\\ {}{b}_2={K}_{\mathrm{A}}{K}_{\mathrm{B}}+{K}_{\mathrm{B}}{K}_{\mathrm{C}}+{K}_{\mathrm{A}}{K}_{\mathrm{C}}\\ {}{b}_3={K}_{\mathrm{A}}{K}_{\mathrm{B}}{K}_{\mathrm{C}}\end{array}} $$

After finding the optimal parameters of the rational function, for instance by curve fitting with the use of the method of the least squares, it is possible to determine the Stern-Volmer quenching constants of particular species as well as the ‘point’ fluorescence intensity fractions assigned to these components. For this purpose one has to solve a system consisting of the following polynomial equations

15 $$ {\displaystyle \begin{array}{l}-{K}_{\mathrm{A}}^3+{b}_1{K}_{\mathrm{A}}^2-{b}_2{K}_{\mathrm{A}}+{b}_3=0\\ {}-{K}_{\mathrm{C}}^2+\left({b}_1-{K}_{\mathrm{A}}\right){K}_{\mathrm{C}}-\frac{b_3}{K_{\mathrm{A}}}=0\\ {}{K}_{\mathrm{B}}={b}_1-{K}_{\mathrm{A}}-{K}_{\mathrm{C}}\end{array}} $$

as well as the matrix equation

16 $$ \left(\begin{array}{c}{a}_1\\ {}{a}_2\\ {}{a}_3\end{array}\right)=\left(\begin{array}{ccc}{K}_{\mathrm{A}}& {K}_{\mathrm{B}}& {K}_{\mathrm{C}}\\ {}{K}_{\mathrm{A}}\left({K}_{\mathrm{B}}+{K}_{\mathrm{C}}\right)& {K}_{\mathrm{B}}\left({K}_{\mathrm{A}}+{K}_{\mathrm{C}}\right)& {K}_{\mathrm{C}}\left({K}_{\mathrm{A}}+{K}_{\mathrm{B}}\right)\\ {}{K}_{\mathrm{A}}{K}_{\mathrm{B}}{K}_{\mathrm{C}}& {K}_{\mathrm{A}}{K}_{\mathrm{B}}{K}_{\mathrm{C}}& {K}_{\mathrm{A}}{K}_{\mathrm{B}}{K}_{\mathrm{C}}\end{array}\right)\left(\begin{array}{c}{f}_{\mathrm{A}}\\ {}{f}_{\mathrm{B}}\\ {}{f}_{\mathrm{C}}\end{array}\right) $$

**Fig. 3:**
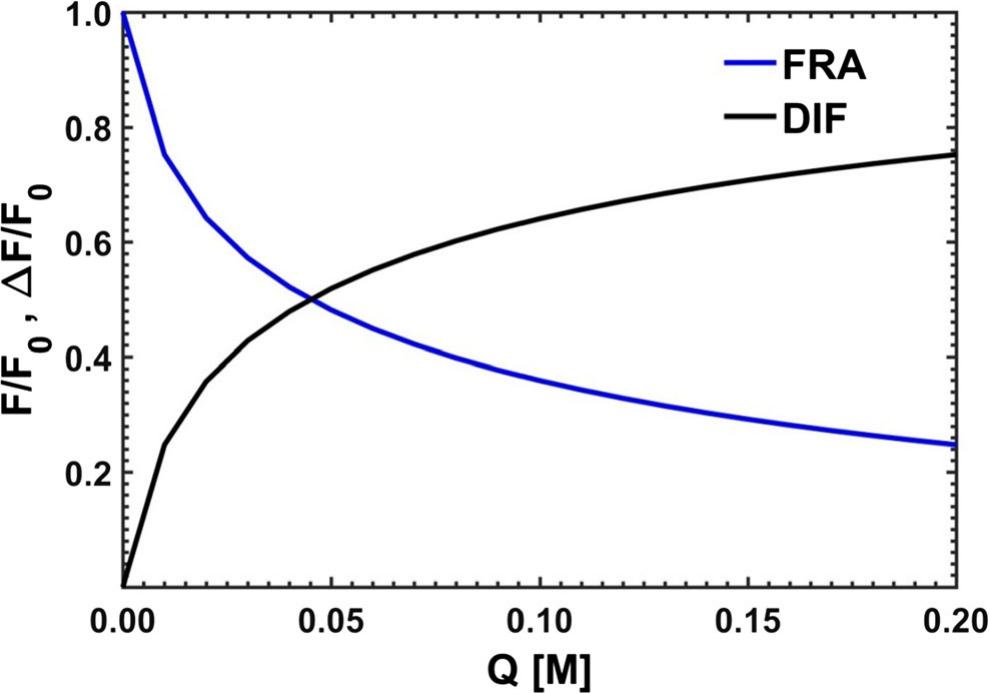
Model curves used to determine the values of Stern-Volmer constants by Lehrer (DIF) and Acuña (FRA) methods

A similar algorithm but operating directly on the apparent parameters of the fitted functional curve was proposed by Acuña et al. [40]. Adopting the following form of the Stern-Volmer dependence

17 $$ \frac{F}{F_0}=\frac{f_{\mathrm{A}}}{1+{K}_{\mathrm{A}}Q}+\frac{f_{\mathrm{B}}}{1+{K}_{\mathrm{B}}Q}+\frac{f_{\mathrm{C}}}{1+{K}_{\mathrm{C}}Q} $$

leads to the sum of three curves, the parameters of which are the Stern-Volmer constants and intensity fractions of particular substances. The applied optimization allows then for determining the required values describing the studied system without need of solving any additional equations (see Fig. 3).

### A Brief Description of the Applied Algorithm

The approaches described above are based on curve fitting with the use of the method of the least squares and therefore it seems quite appropriate to briefly quote what are the operating principles of one commonly used optimization algorithm, i.e. a Newton-Gauss algorithm with a Levenberg-Marquardt extension, as explained by Maeder and Neuhold [41]. The cited procedure is based on minimization of the difference, **r**, between the real data given in a form of a vector, **y**, and the data resulting from the optimal functional form, **y**_opt_

18 $$ \mathbf{r}\left(\mathbf{p}\right)=\mathbf{y}-{\mathbf{y}}_{\mathrm{opt}}\left(\mathbf{p}\right) $$

As it can be noticed the above difference depends on parameters **p** of the fitted function, thus by changing the vector of initial parameters by a certain value, δ**p**, it is possible to obtain the error vector **r**(**p** + δ**p**) with smaller elements in the least-squares sense (the sum of squares *ssq* = **r**^**T**^**r** should be minimal, or at least smaller), optimally equal zero. The residuals **r**(**p** + δ**p**) are approximated by a Taylor series expansion

19 $$ \mathbf{r}\left(\mathbf{p}+\updelta \mathbf{p}\right)=\mathbf{r}\left(\mathbf{p}\right)+\frac{\partial \mathbf{r}\left(\mathbf{p}\right)}{\partial \mathbf{p}}\left[\left(\mathbf{p}+\updelta \mathbf{p}\right)-\mathbf{p}\right]+\dots $$

which upon retaining the first two terms and introducing a Jacobi matrix of the first partial derivatives gives

20 $$ 0\approx \mathbf{r}\left(\mathbf{p}+\updelta \mathbf{p}\right)=\mathbf{r}\left(\mathbf{p}\right)+\mathbf{J}\updelta \mathbf{p} $$

which eventually upon simple transformation using the idea of pseudoinversion leads to the matrix equation that allows to determine the ‘best’ parameter shift vector δ**p**

21 $$ \updelta \mathbf{p}=-{\mathbf{J}}^{+}\mathbf{r}\left(\mathbf{p}\right) $$

Upon performing a simple operation of addition of two vectors

22 $$ {\mathbf{p}}^{'}=\mathbf{p}+\updelta \mathbf{p} $$

a better convergence, at least on the theory grounds, between the real and optimized functions is achieved.

**Fig. 4:**
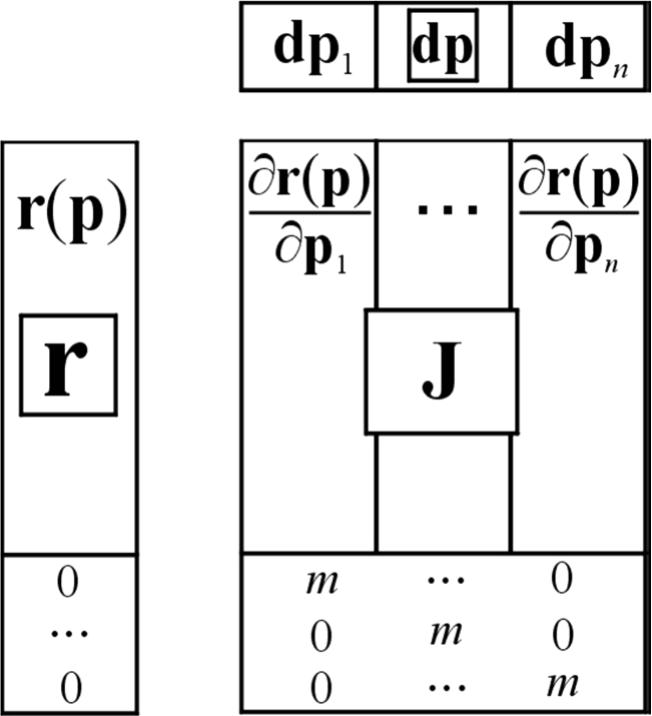
Matrix illustration of the nglm algorithm

Sometimes, however, the input values of parameters **p** depart significantly from optimal values – in such case the Levenberg-Marquardt extension to the Gauss-Newton minimizer can be used to ‘protect’ the algorithm from taking a too big step or inappropriate direction. This correction consists in ‘elongation’ of the error vector by an appropriate amount of zero rows and augmentation of the Jacobi matrix, **J**, by a diagonal matrix with all the elements on the diagonal equal to a predefined value *m* (see Fig. 4). A numeric value of the Marquardt parameter *m* is not uniquely determined and should be suitably adjusted in each optimization case. The detailed description of the construction of this algorithm goes far beyond the contents of this article, hence the Reader is suggested to refer to other Literature dealing with this particular issue.

### Matrix Representation of Stern-Volmer Profiles

The optimization methods discussed above allow for taking into account the fluorescence intensity at only one emission wavelength. Of course, it is possible to carry out a series of individual optimizations for all emission wavelengths, however, the obtained Stern-Volmer constants of a given fluorophore that theoretically should be equal, remain actually independent which may lead to a few hundreds of different values depending on a measuring point, *λ*. To solve this problem one has either to take an average or to get down to constructing some matrix versions of the optimization algorithm, which form the basis for the modern methods of the multivariate curve resolution.

The ‘white’ algorithms are directly based on the matrix factorization illustrated in Fig. 1 and described by Eq. (2). The resulting **S**^**T**^ (see Eq. (3)) stems from the ‘concentration’ matrix which is constructed on the basis of the used concentrations of a quencher and some preliminary initialized Stern-Volmer quenching constants, **K**.

23 $$ \mathrm{C}\left(Q,n\right)=\frac{1}{1+{K}_nQ} $$

In the above formula if *Q* = 0 then C(0, *n*) = 1, so the number of rows in matrix **C** formed by *n* Stern-Volmer profiles is actually by 1 greater than the number of different concentrations of the quencher. The matrix, **Y**_opt_ = **CS**^**T**^, is subsequently used in the nglm algorithm.

24 $$ \mathbf{r}\left(\mathbf{K}\right)=\mathbf{Y}-{\mathbf{Y}}_{\mathrm{opt}}\left(\mathbf{K}\right)=\mathbf{Y}-{\mathbf{CS}}^{\mathbf{T}} $$

By introducing corrections to matrix **C** which are brought about only by the change in the values of the Stern-Volmer quenching constants, a better conformity between the empirical data collected in matrix **Y** and the data contained in matrix **Y**_opt_ is achieved. Finally, as a result of the optimization process both the quenching constants and spectral profiles are obtained, on the basis of which the best description of the studied system can be proposed.

While in ‘classical’ approach to decomposition of multi-component spectra it is assumed that the recorded spectra of quenched fluorescence are inserted into a data matrix in their ‘unaltered’ form, yet in an approach that makes use of the idea of ‘spectral fractions’ this natural assumption is modified. The method takes advantage of a notably different form of the data matrix which is actually an extension of the original ‘point’ methods as proposed by Lehrer [39] and Acuña et al. [40]. The original data matrix **Y** is replaced by a matrix **Y**_**f**_ in which the elements of each row are obtained by point by point division by the corresponding elements of the first raw of the original data matrix.

The matrix equation on which the mathematical operations of the applied algorithm are performed remains unchanged, however, as a result of the optimization procedures the fractions of the overall fluorescence intensity, **f**, instead of the emission profiles are obtained.

25 $$ {\mathbf{f}}^{\mathbf{T}}={\mathbf{C}}^{+}{\mathbf{Y}}_{\mathrm{f}} $$

Matrix **f** is sized $\left(\lambda\ x\ \mathtt{n}\right)$ where *λ* is the number of emission wavelengths, and *n* is the number of significant components. In the case of a three-component system (*n* = 3), the spectral fractions at each specified emission wavelength when summed up, give one, i.e. *f*^*A*^ + *f*^*B*^ + *f*^*C*^ = 1. The size of **S** is also $\left(\lambda\ x\ \mathtt{n}\right)$. Thus, the transformation from **f** to **S** is performed for each matrix entry using the first fluorescence spectrum, **y**_0_ = **Y**(1, *λ*), measured in the absence of the quencher.

26 $$ {s}_{ij}={f}_{ij}{y}_{0,i}\kern1.75em \left(i=1,\dots, \lambda; \kern1.25em j=1,\dots, n\right) $$

In Eq. (26) *y*_0, *i*_ represents the *i*-th element of vector **y**_0_.

The methods described above are classified as ‘white’ methods because of the assumption concerning the fulfillment of the linear Stern-Volmer equation. Despite the useful approximation provided by a chemical model, the hidden disadvantage carried by hard methods are, in the considered case, the values of the quencher concentration assumed to be absolutely constant. However, it is well-known that even the best measurement procedure is endowed with uncertainties and therefore, as regards the assumed values of *Q*, some almost imperceptible departures are unavoidable. The solution to this problem may be provided by so called ‘grey’ methods of data modeling that do not impose stiff constraints on the amount of the quenching substance contained in a sample.

The ‘hard-soft’ methods of data modeling incorporate advantages of both the methods obeying the restrictive criteria of ‘white’ methods and the ‘black’ procedures void of any constraints except for non-negativity. This approach seems to combine two things that are mutually exclusive but there is no contradiction as it has been proven on the example of the MCR ALS (*Multivariate Curve Resolution Alternating Least Squares*) algorithm elaborated by Tauler et al. [42, 43].

Likewise in the case of the discussed ‘hard’ methods, the ‘grey’ (hard-soft) algorithm is operating on three matrices: original data matrix containing the measured multi-component spectra, **Y**, and with regard to particular components, the matrix of the fluorescence intensity decays (‘concentration’ matrix), **C**, and the matrix of spectral profiles, **S**^**T**^. The first step is analogous: the matrix **C** is built on the basis of known concentrations of the quencher and tentatively determined Stern-Volmer constants (this stands for the ‘white’ element). Then the initial matrix **S**^**T**^ and the trial matrix **Y**_opt_ are generated. In the next step, however, a significant difference emerges: the concentration matrix is no more optimized only on the basis of the quenching constants, but by itself as a whole constitutes a parameter which undergoes a permanent optimization and adaptation process (this represents the ‘black’ element). To avoid the values without physical meaning the non-negativity constraint (a ‘white’ element) becomes superimposed on the profiles in matrices **C** and **S**^**T**^. Eventually, a pair of vectors, **c**_*n*_ and **s**_*n*_, representing the emission intensity decay and the spectral profile (a spectrum) of a given component *n*, is generated.

## Experimental

**Fig. 5:**
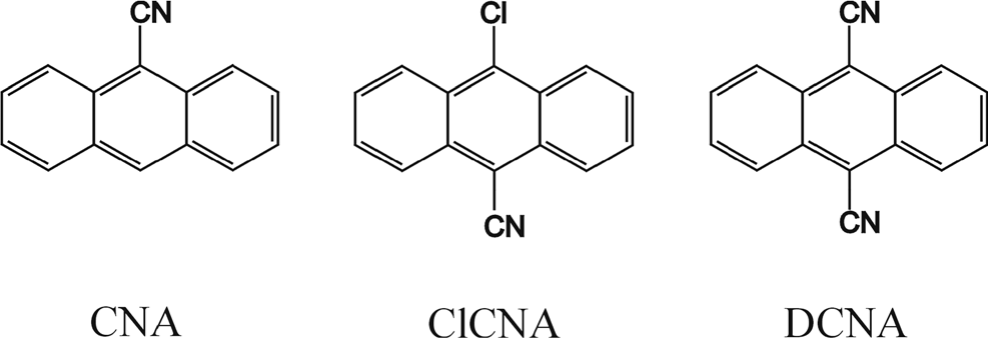
Fluorophores used in this study

**Fig. 6:**
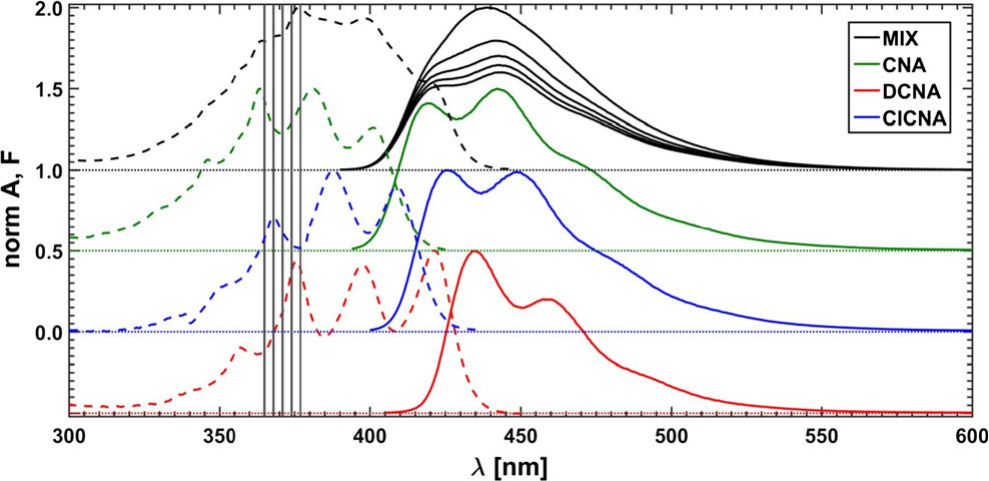
Measured absorption (dashed lines) and emission (solid lines) of pure components and of the mixture (MIX); vertical lines mark the selected excitation wavelengths

As first the absorption and emission spectra of three pure fluorophores: 9-cyanoanthracene (CNA), 9,10-dicyanoanthracene (DCNA) and 10-chloro-9-cyanoanthracene (ClCNA) (see Fig. 5) were measured in methanol solutions using a Hitachi U-2900 spectrophotometer and a Hitachi F7000 fluorimeter, respectively (see Fig. 6). Then, in order to minimize the inner filter effect the mixtures were diluted, so that the absorption maximum of each spectrum was lower than 0.04.

**Fig. 7:**
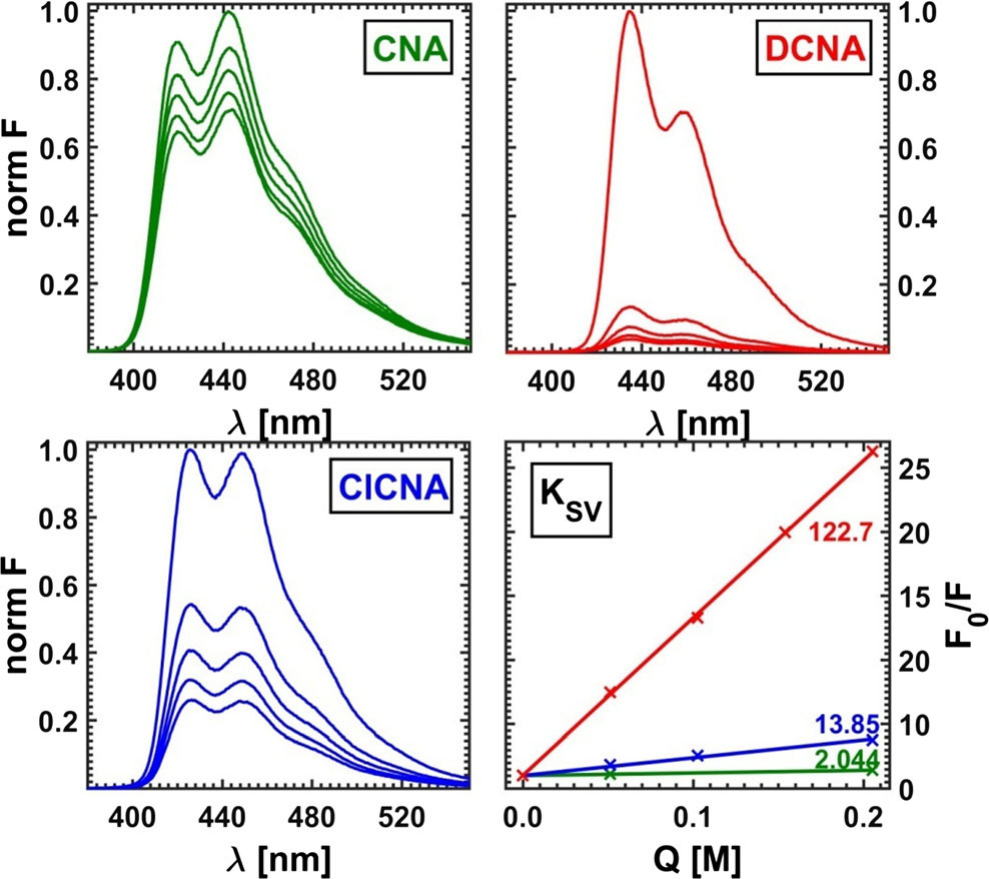
Quenched fluorescence spectra of pure substances: **a** CNA, **b** DCNA, **c** CICNA and **d** the related Stern-Volmer plots

In order to determine the Stern-Volmer constants of pure fluorophores, the quenched fluorescence spectra were measured for a series of samples using the previously prepared CNA, DCNA and ClCNA solutions with linearly increasing amounts of lithium bromide added as a quencher up to the highest concentration of 0.2 M. The values of the fluorescence intensities of subsequent samples at the wavelength for which the intensity of the unquenched emission was the highest were plotted versus the concentration of a quencher. Next, for each substance the Stern-Volmer straight line was fitted to the calculated data points and the related quenching constant was extracted (see Fig. 7).

Finally, a mixture of all three fluorophores was made. The concentrations were chosen in such a way that the absorption maximum for each substance was below 0.08. 21 equal portions of the solution were taken and the increasing aliquots of the quencher solution (lithium bromide in methanol) were added. To prevent appearance of a possible interference of Rayleigh and Raman scattering, inevitably associated with the excitation lines, these were carefully selected far away from the range of the recorded fluorescence spectra at five fixed wavelengths: 365, 368, 371, 374 and 377 nm. Moreover, it goes without saying, as regards the mixture of fluorophores, that varying of the excitation wavelength is responsible for (leads to) the change in the relative amounts of the fluorescence emitting species. The typical measured spectra of the unquenched mixture as well as individual components are presented in Fig. 6. Additionally, the absorption spectra were recorded for two outermost samples: without the quencher and with the highest quencher concentration. The concentrations of the used fluorophores and lithium bromide expressed in M (mole/dm^3^) were as follows:

**Table:** 

c_CNA_ = 7.68 ⋅ 10^−6^	c_DCNA_ = 1.84 ⋅ 10^−5^
c_ClCNA_ = 9.47 ⋅ 10^−6^	c_LiBr_ = 0, 0.0103, 0.0206, …, 0.2056

**Fig. 8:**
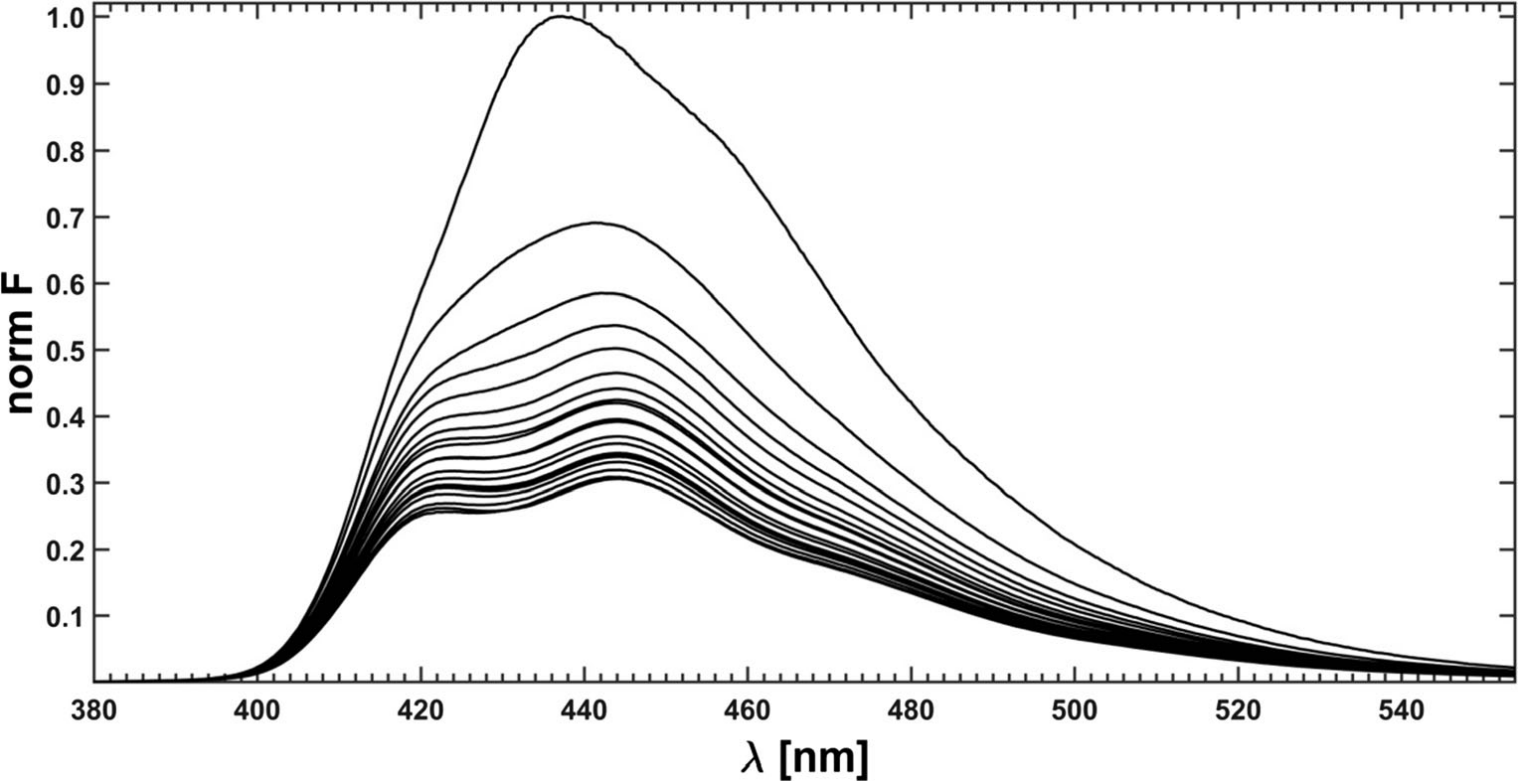
Quenched fluorescene spectra of fluorophore mixture after preprocessing; excitation line = 368 nm

The recorded raw spectra were preprocessed: the methanol (solvent) baseline was subtracted, correction for self-absorption (inner filter effect) was made and the data reproduction using the SVD procedure, producing the ‘smoothed’ data, was applied (if not, then it is marked in the text). A series of such spectra upon preprocessing is shown in Fig. 8.

## Results and Discussion

### Synthetic Data

**Fig. 9:**
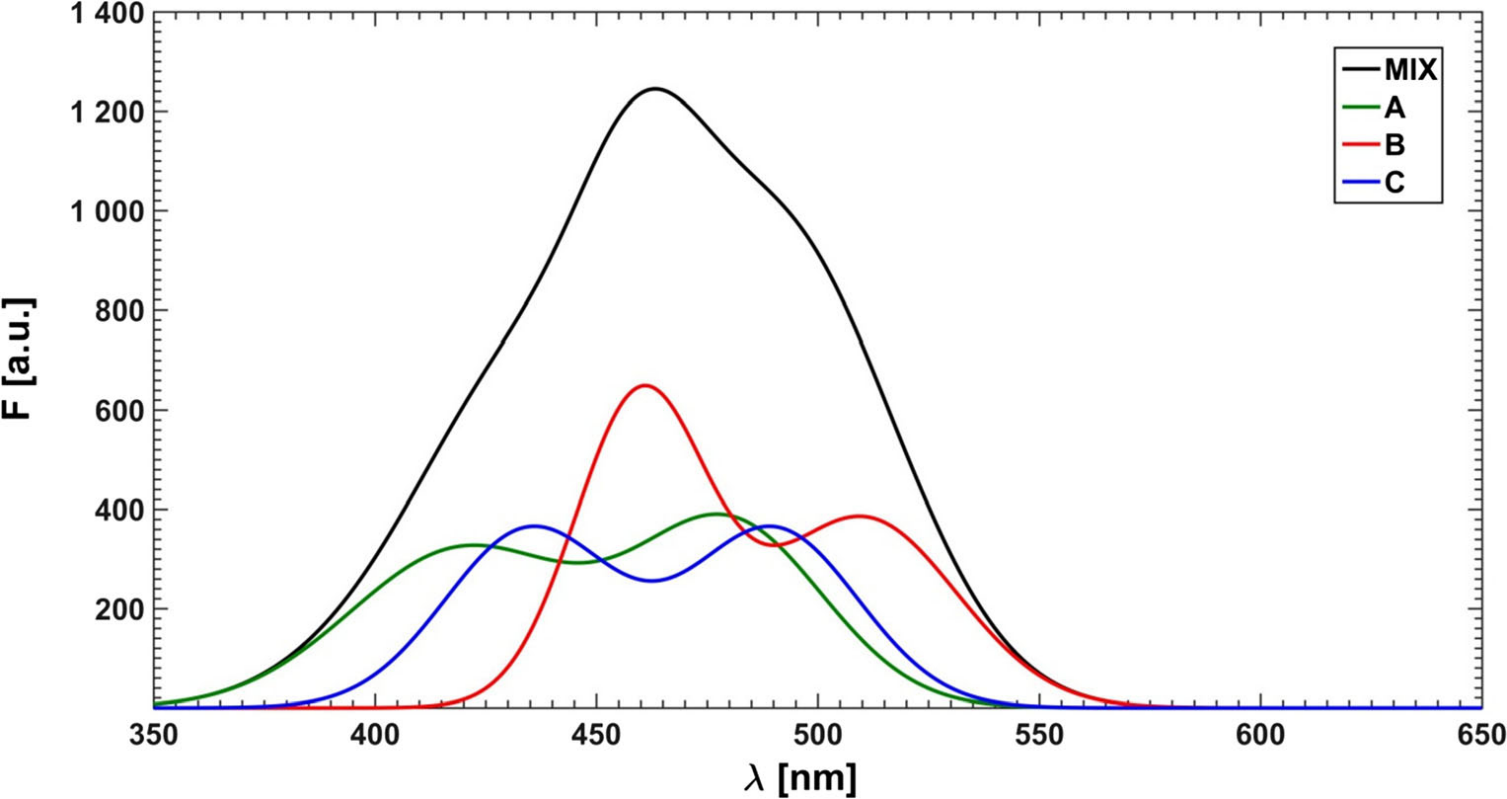
Simulated fluorescence spectra of single components A, B and C summed up into a mixture spectrum (MIX)

All required data processing, calculations and analyses for this study were performed with MATLAB R2012a (The MathWorks, Inc., Natick, MA) software. For practical reasons, in some cases the Authors have taken the liberty of keeping the unchanged MATLAB notation. A model system of quenched fluorescence of three components: A, B and C was simulated (see Fig. 9) by using a set of a few Gaussian envelopes, partially based on real spectra. Stern-Volmer constants were defined as follows:

$$ {\mathtt{K}}_{\mathtt{A}}=\mathtt{5.00}\kern0.2em {\mathtt{M}}^{-\mathtt{1}}\kern0.5em {\mathtt{K}}_{\mathtt{B}}=\mathtt{1}\mathtt{00}\kern0.2em {\mathtt{M}}^{-\mathtt{1}}\kern0.5em {\mathtt{K}}_{\mathtt{C}}=\mathtt{20.0}\kern0.2em {\mathtt{M}}^{-\mathtt{1}} $$

or in MATLAB notation: K = [5.00; 100; 20.0].

**Fig. 10:**
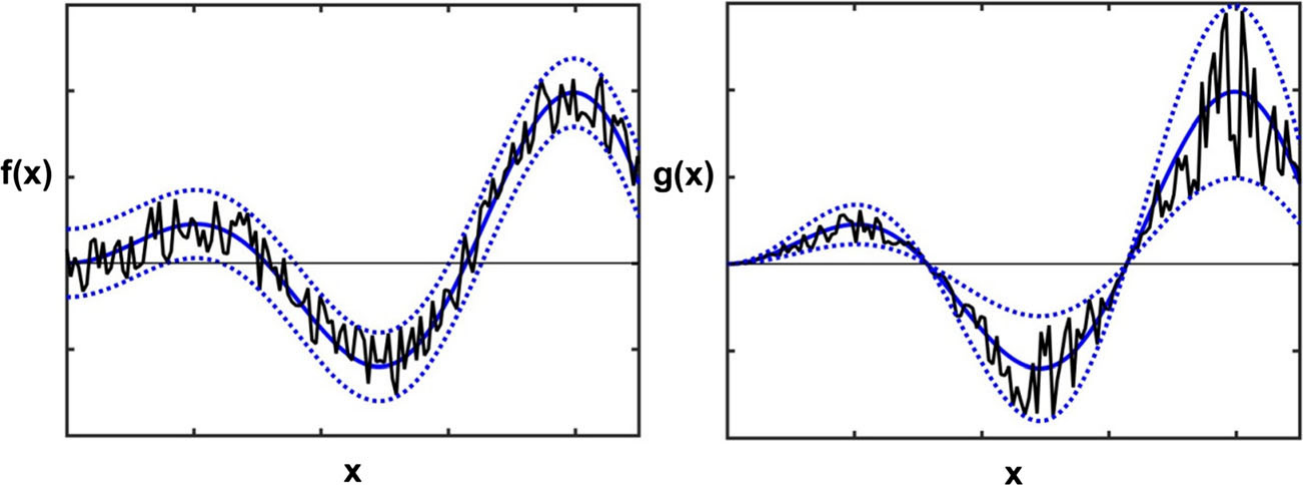
Two types of data noise: **a** additive (independent of signal) and **b** multiplicative (proportional to signal)

Next, on the basis of the linear Stern-Volmer equation, a series of 21 three-component spectra was formed, each one simulating fluorescence at particular quencher concentration – linearly increasing from 0 to a value of 0.2 M. In order to imitate real dataset, two types (see Fig. 10) of data noise were added to these ideal spectra. The first one, marked as S – fixed or additive noise (known also as homoscedastic noise) – represents instrumental type of noise, which is independent of the magnitude of a signal (in this case independent of the response of a fluorimeter). The second noise is a variant of the heteroscedastic noise [44], denoted as Q –and is linearly proportional to a variable - in this case to a quencher concentration; it simulates an imperfection in the amounts of substances used to prepare a sample. Because those two types of data noise are different, their noise level is defined distinctly as:

$$ {\displaystyle \begin{array}{l}\mathrm{fixed}:\kern0.5em n\left(\lambda \right)=F\left(\lambda \right)+y\cdot r\cdot \max (F)\\ {}\begin{array}{cc}\mathrm{proportional}:& n(Q)=Q+x\cdot r\cdot Q\end{array}\end{array}} $$

where: *n* – ‘noisy’ data, *r* – random numbers from −1 to 1, *F*(*λ*) – fluorescence intensity at wavelength *λ*, max(*F*) – the highest value of fluorescence intensity in a series, *Q* – quencher concentration, *y* – spectral data noise level (in ‰), *x* – concentration data noise level (in %). To conclude: the notation *Qx* or *Sy* stands for: *Q, S* – type of noise; *x, y* – data noise level.

**Fig. 11:**
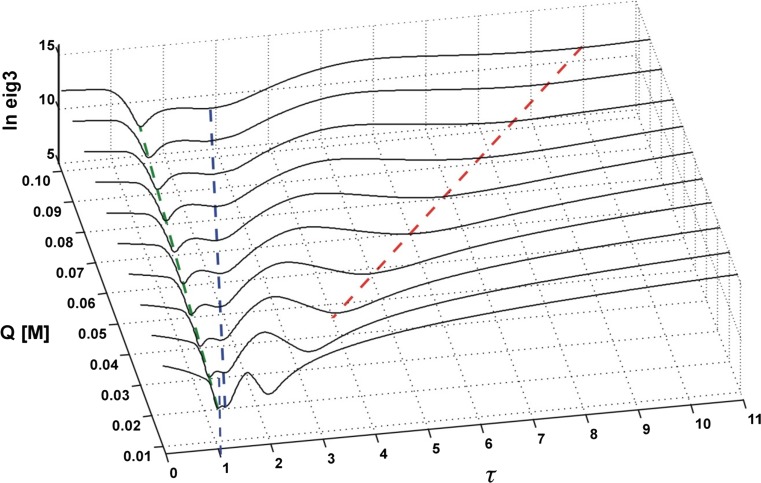
*τ*-RAFA applied to simulated data; data noise level Q5%, S05‰

**Fig. 12:**
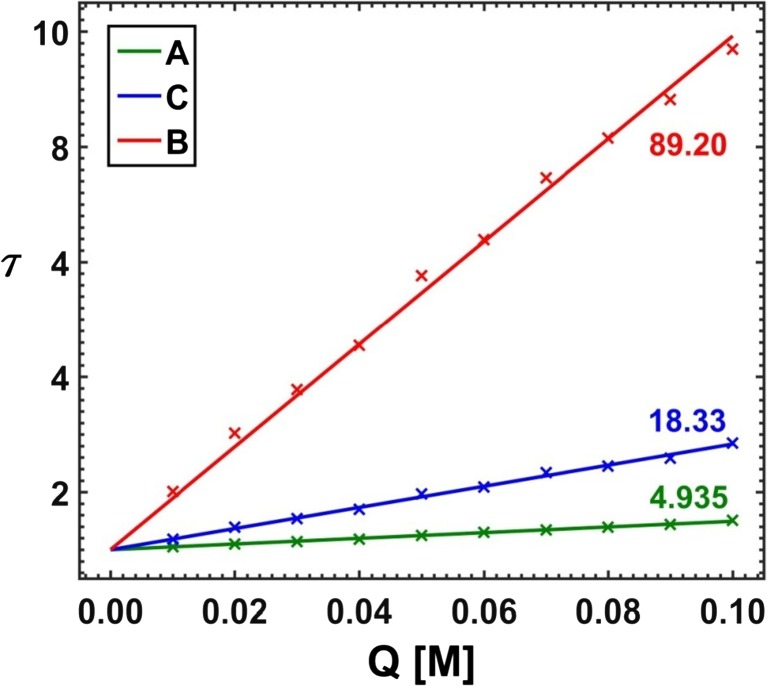
Stern-Volmer plots based on results of *τ*-RAFA; data noise level Q5%, S05‰

**Fig. 13:**
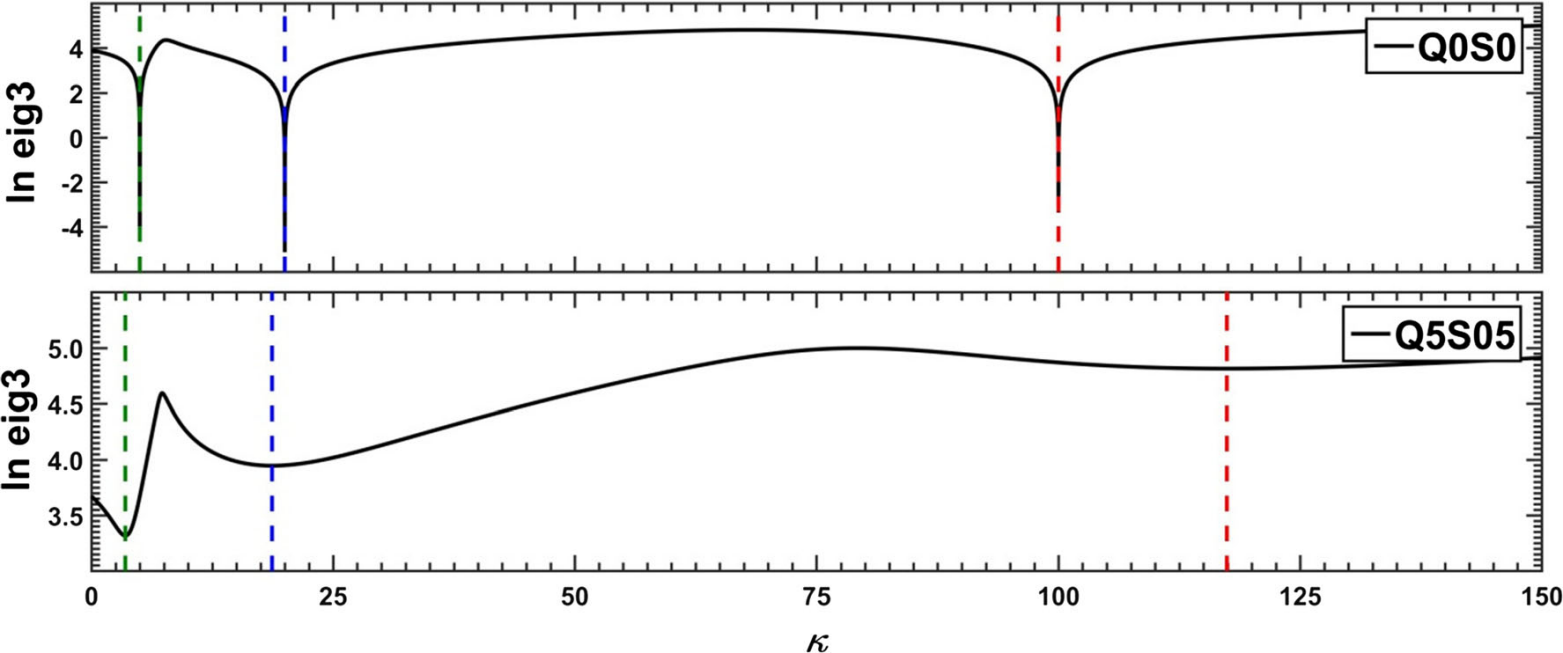
Stern-Volmer constants determined by application of ‘direct’ *κ*-RAFA to model data: **a** without noise, and **b** with noise level Q5%, S05‰

In order to faithfully reflect the nature of the applied analytical procedure, the analysis of the simulated system was commenced with estimation of the Stern-Volmer constants of all three substances A, B and C. To this purpose both versions of the RAFA technique (*τ*‐RAFA and *κ*‐RAFA, see “Rank Annihilation Factor Analysis” Subsection) were used. Furthermore, at the same time, an influence of data type and noise level on the final results was also investigated – the findings are presented in Figs. 11, 12, and 13 and in Table 1. On the basis of the performed research, it can be said that the faster *κ*-RAFA procedure remains more sensitive to noisy data and the calculated values are remarkably divergent from expected ones. The accuracy of more complex *τ*-RAFA approach is higher, however, reliable application of both methods is limited by the noise level – predominantly instrumental, and therefore the margin for an acceptable error should be kept below S1‰.

**Table 1:** Stern-Volmer constants of individual components determined by two versions of RAFA for different noise types and levels

‘Noise’	*τ*-RAFA			*κ*-RAFA		
	K_A_	K_C_	K_B_	K_A_	K_C_	K_B_
Q0S0	5.00	20.0	100	5.00	20.0	100
Q3	5.01	19.9	100	4.24	20.4	100
Q5	5.13	20.5	103	3.54	19.2	120
Q10	5.19	20.7	104	2.44	18.7	138
S03	4.89	19.2	94.1	4.86	17.4	91.7
S05	4.81	18.4	88.3	4.76	16.0	86.9
S1	4.51	–	71.7	4.22	12.9	74.7
Q3S03	4.89	19.0	93.2	4.22	20.0	100
Q5S05	4.94	18.3	89.2	3.50	18.7	117

^a^Q in % and S in ‰

^b^S-V constants in M

The next interesting issue addressed by the Authors was to assess the performance and outcome of the ‘point’ optimization methods as proposed by Lehrer [39] and Acuña et al. [40] in the form of third-order rational function and the sum of three rational functions of the first degree, respectively. In the first case, the use of the nglm optimization algorithm results in ambiguity when the respective terms, containing *Q* as variable, in the formula shown below attain values substantially greater than 1. Then the constant term in the denominator – equal to 1 – begins to lose its significance and the parameters *a* and *b* can be multiplied by any factor whatsoever, which may lead to huge orders of magnitude and lack of the optimization progress.

27 $$ \mathrm{g}(Q)=\frac{a_1Q+{a}_2{Q}^2+{a}_3{Q}^3}{1+{b}_1Q+{b}_2{Q}^2+{b}_3{Q}^3} $$

One of the suggested solutions was to make a variable out of the constant in the denominator.

28 $$ \mathrm{g}(Q)=\frac{a_1Q+{a}_2{Q}^2+{a}_3{Q}^3}{p+{b}_1Q+{b}_2{Q}^2+{b}_3{Q}^3} $$

Theoretically, it would allow to avoid extremely large orders of magnitude by dividing all the coefficients *a* and *b* by a variable parameter *p* (*p*/*p* = 1). Unfortunately, that train of thought is not compatible with the MATLAB nglm algorithm, since the shift vector applied during the optimization process is responsible merely for the increase of the parameters *a* and *b* but does not change their ratios. Another possible approach was to transform the original mathematical formula, Eq. (27), into the following:

29 $$ \mathrm{g}(Q)=\frac{\frac{a_1}{a_3}Q+\frac{a_2}{a_3}{Q}^2+{Q}^3}{\frac{1}{a_3}+\frac{b_1}{a_3}Q+\frac{b_2}{a_3}{Q}^2+{Q}^3}=\frac{p_1Q+{p}_2{Q}^2+{Q}^3}{p_3+{p}_4Q+{p}_5{Q}^2+{Q}^3} $$

The equality of parameters *a*_3_ and *b*_3_ results from their entanglement with the Stern-Volmer constants (see “Theoretical Background”)

30 $$ {a}_3={K}_{\mathrm{A}}{K}_{\mathrm{B}}{K}_{\mathrm{C}}\left({f}_{\mathrm{A}}+{f}_{\mathrm{B}}+{f}_{\mathrm{C}}\right)={K}_{\mathrm{A}}{K}_{\mathrm{B}}{K}_{\mathrm{C}}={b}_3 $$

Unfortunately, also that method did not provide a solution to the encountered optimization problems. Finally, a decision was arrived at to use the original third-order rational formula, but with the number of coefficients reduced to five (*a*_3_ = *b*_3_). This approach turned out to be only partly successful. Apparently, it appears that the form most suitable for use by the optimizer is the sum of three first-degree rational functions. A problem of ambiguity of solutions is eliminated and so are additional calculations required to obtain the Stern-Volmer constants and spectral fractions from the optimized parameters (see “Theoretical Background”).

In order to compare the efficiency of the two ‘point’ methods of optimization mentioned above, all calculations were related to the same simulated spectral dataset. Also, the same initial values of spectral fractions and Stern-Volmer constants were used in both cases:

$$ \mathbf{f}=\left[0.3000;0.3333;0.3337\right]\kern0.5em \mathbf{K}=\left[4.00;15.0;120\right] $$

According to the Authors’ assumptions, all initial spectral fractions should be equal, but the nglm algorithm in MATLAB requires at least slightly different starting values – if they are identical, the procedure simply does not work properly.

**Fig. 14:**
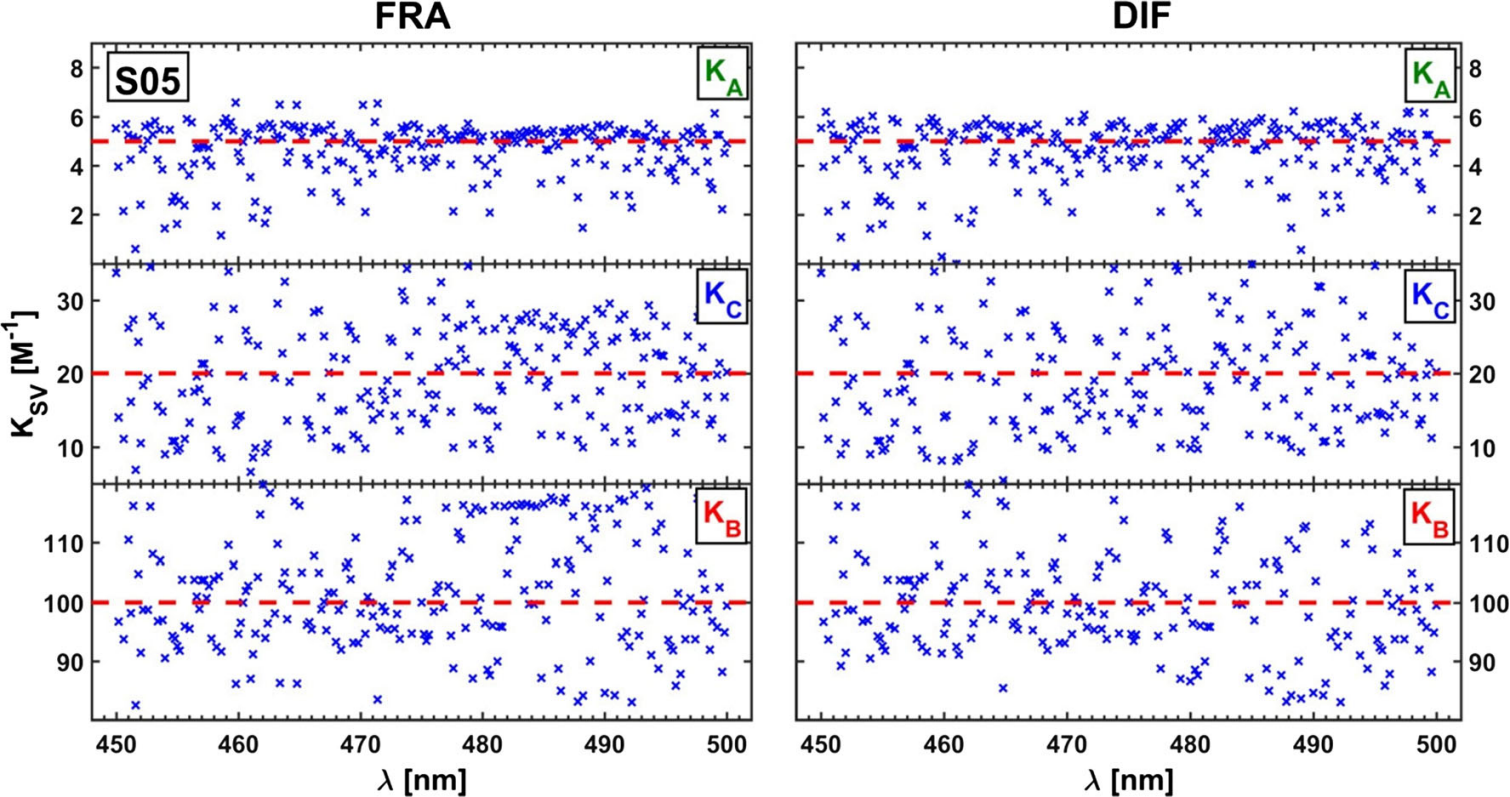
Characteristic spreads of Stern-Volmer constants determined by ‘point-by-point’ optimization using the methods of Acuña et al. [40] - **FRA**, and Lehrer [39] – **DIF**

Due to a divergence of the results (see Fig. 14), obtained independently for different emission lines, a mean of all the optimized point-by-point parameters was calculated (FRA – sum of three hyperbolas, DIF – third degree rational):

$$ {\mathbf{K}}_{FRA}=\left[4.67;24.8;105\right]\kern0.5em {\mathbf{K}}_{DIF}=\left[4.60;23.7;115\right] $$

**Fig. 15:**
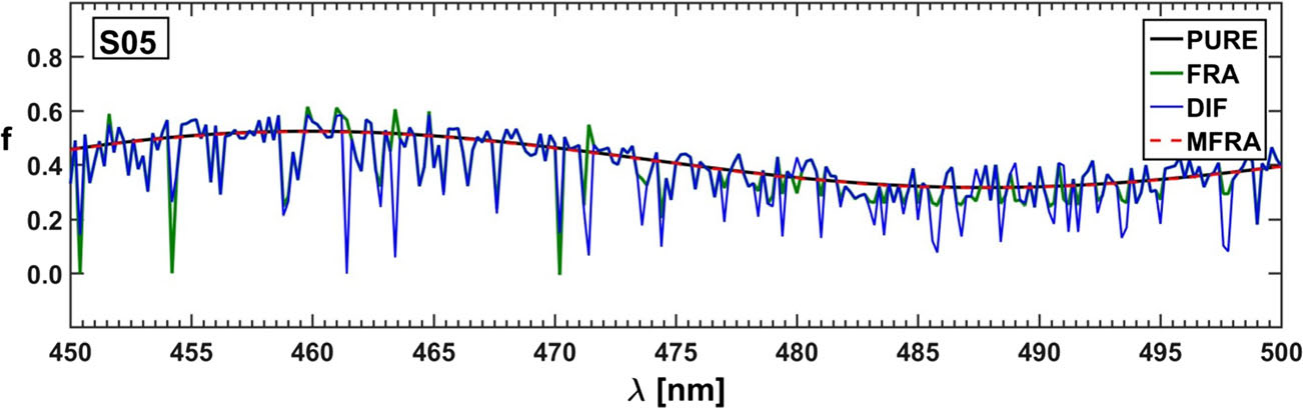
Spectral fractions for simulated component B: PURE – expected curve shape, FRA - Acuña et al. method [40], DIF – Lehrer method [39], and MFRA - Acuña et al. approach in matrix version

The computed Stern-Volmer constants are somewhat more convergent with the expected ones than the constants obtained by the use of the *τ*− and *κ*-RAFAmethods. Moreover, the application of both Lehrer and Acuña approaches allowed to determine spectral fractions of all three substances for almost each selected emission line, though the fractional profiles reconstructed on their basis could be described as ‘rugged’ and discontinuous (see Fig. 15). In conclusion, it can be noted that the ‘point’ methods were historically justified. Fitting one rational function was easier than fitting a sum of three hyperbolic curves, but nowadays a complexity of a fitted combinations of formulas has become less problematic.

On the basis of the averaged values of the Stern-Volmer constants it is possible to restore the concentration matrix **C**, which then can be used to resolve the multi-component fluorescence spectra into the emission profiles of pure substances. Nevertheless, another step ahead can be made, since the ‘point-by-point’ optimization can readily be replaced by the optimization performed as a whole on full data matrices.

All white methods of data modeling that have been exploited in this article make use of the same form of the concentration matrix **C** containing the individual intensity decays recovered on the basis of the preliminary estimated values of the Stern-Volmer constants. Therefore it seemed appropriate to investigate the influence of initial parameters on final results obtained through application of the nglm algorithm. A series of different initial vectors have been optimized and the available findings are collected in Table 2. The carried out analysis has proven that the white (or hard) method based on a full data matrix, unlike ‘point’ methods, is practically independent of the user-entered starting values. A concluding statement can be made that the mentioned above independence is likely a result of the increased ratio of two quantities: the number of the data entries to the number of the modified parameters: 21 versus 5 in case of the applied ‘point-by-point’ method and more than 21 × 1000 versus 3 in case of the employed matrix method.

**Table 2:** Stern-Volmer constants of single components before and after optimization on simulated data with assumed noise level Q5%S05‰

Initial			Optimized		
5.00	20.0	100	4.487	19.57	123.6
4.75	19.0	100	4.486	19.57	123.5
4.50	18.0	110	4.486	19.57	123.5
4.00	15.0	120	4.485	19.56	123.5
49.0	50.0	51.0	4.485	19.56	123.5

^a^**)** S-V constants in M

**Fig. 16:**
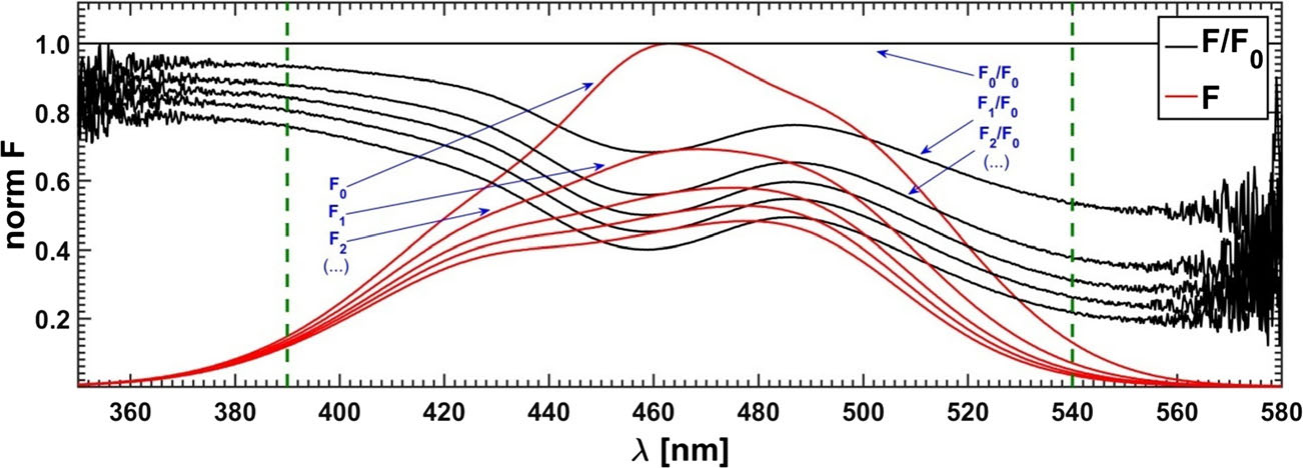
Two forms – classical and fractional of simulated data used in optimizations. Vertical lines confine the spectral region with reduced data noise

**Fig. 17:**
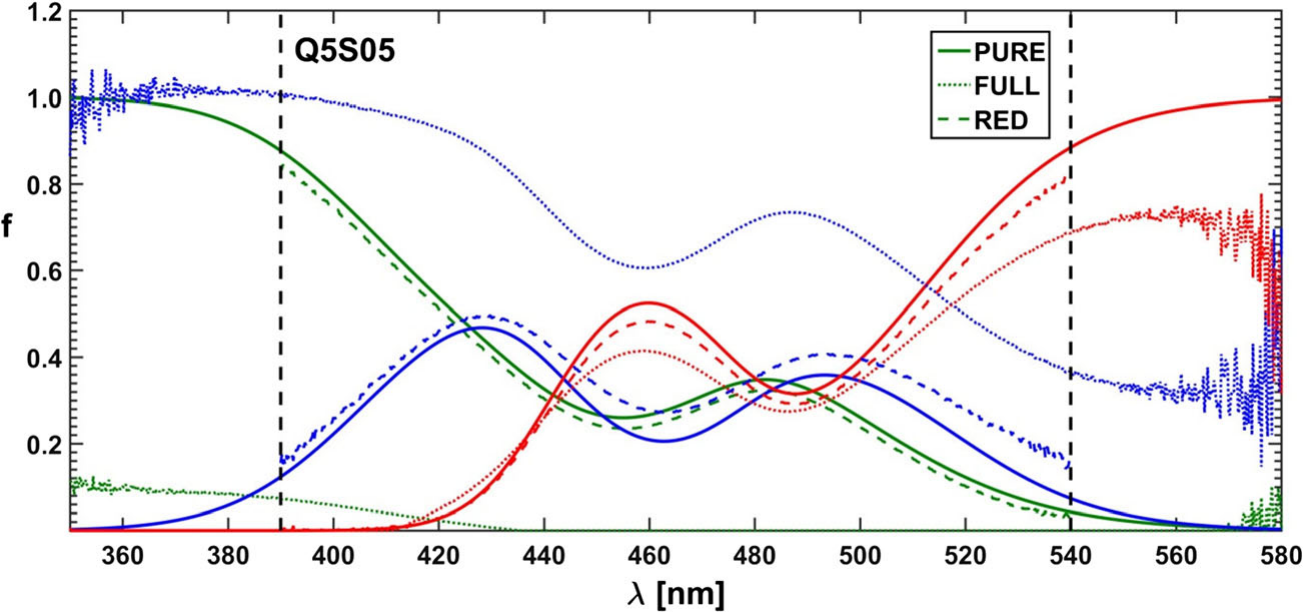
Effect of reduction of data range on the shape of the resolved spectra: PURE – expected curve shape, FULL – complete data range, and RED – data range reduced to the region between vertical lines; data noise level Q5%, S05‰

The difference between ‘classical’ and originated by Acuña et al. ‘fractional’ approach to hard resolution of multi-component spectra lies in a form of data to-be-optimized. In the former case, a dataset is created explicitly from the measured quenched fluorescence spectra, while in the latter case all spectral intensities of a given spectrum are divided ‘point-by-point’ by corresponding intensities of the fluorescence spectrum recorded for the sample without the quencher. An advantage of such normalized data is that the areas of high and low noise level are now easily recognizable (see Fig. 16). If the range of the analyzed spectral data is narrowed to its less noisy portion, the accuracy of the whole procedure is appreciably improved (Fig. 17). Moreover, it is possible to use the concentration matrix optimized this way to reproduce completely resolved spectra of pure components. As a result of application of the ‘fractional’ method the spectral fractions instead the spectra are obtained – emission profiles could then be determined in at least two equivalent ways: by dividing the spectral data matrix ‘point-by-point’ by the resulting concentration matrix or by multiplying the measured spectrum of unquenched fluorescence of the fluorophore mixture ‘point-by-point’ by the fractional profiles.

Finally, a few series of simulated three-component quenched fluorescence spectra with different type and noise level were resolved using ‘classical’ hard method of data modeling, ‘fractional’ white method with reduced spectral range as well as MCR-ALS approach i.e. a grey method of Tauler et al. [42, 43]. The initial concentration matrix was the same in all approaches and it was constructed assuming the following values of the Stern-Volmer constants:

$$ \mathbf{K}=\left[4.00;120;15.0\right] $$

**Fig. 18:**
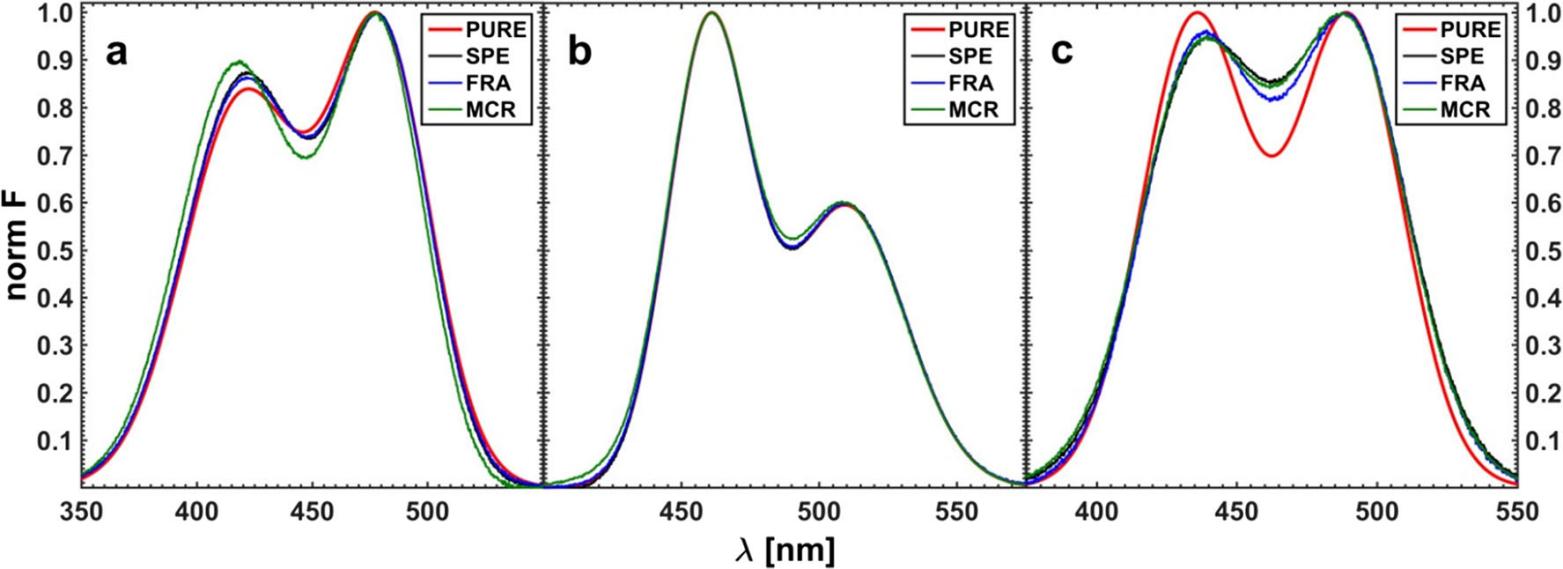
Resolved fluorescence spectra of pure components obtained for the simulated data noise level Q5%, S05‰. PURE – expected curve shape, SPE – obtained by ‘classical’ approach, FRA – yielded using fractional data, and MCR – produced by grey MCR-ALS method of Tauler et al. [42]

The spectra of pure components resolved by the applied methods are depicted in Fig. 18.

**Fig. 19:**
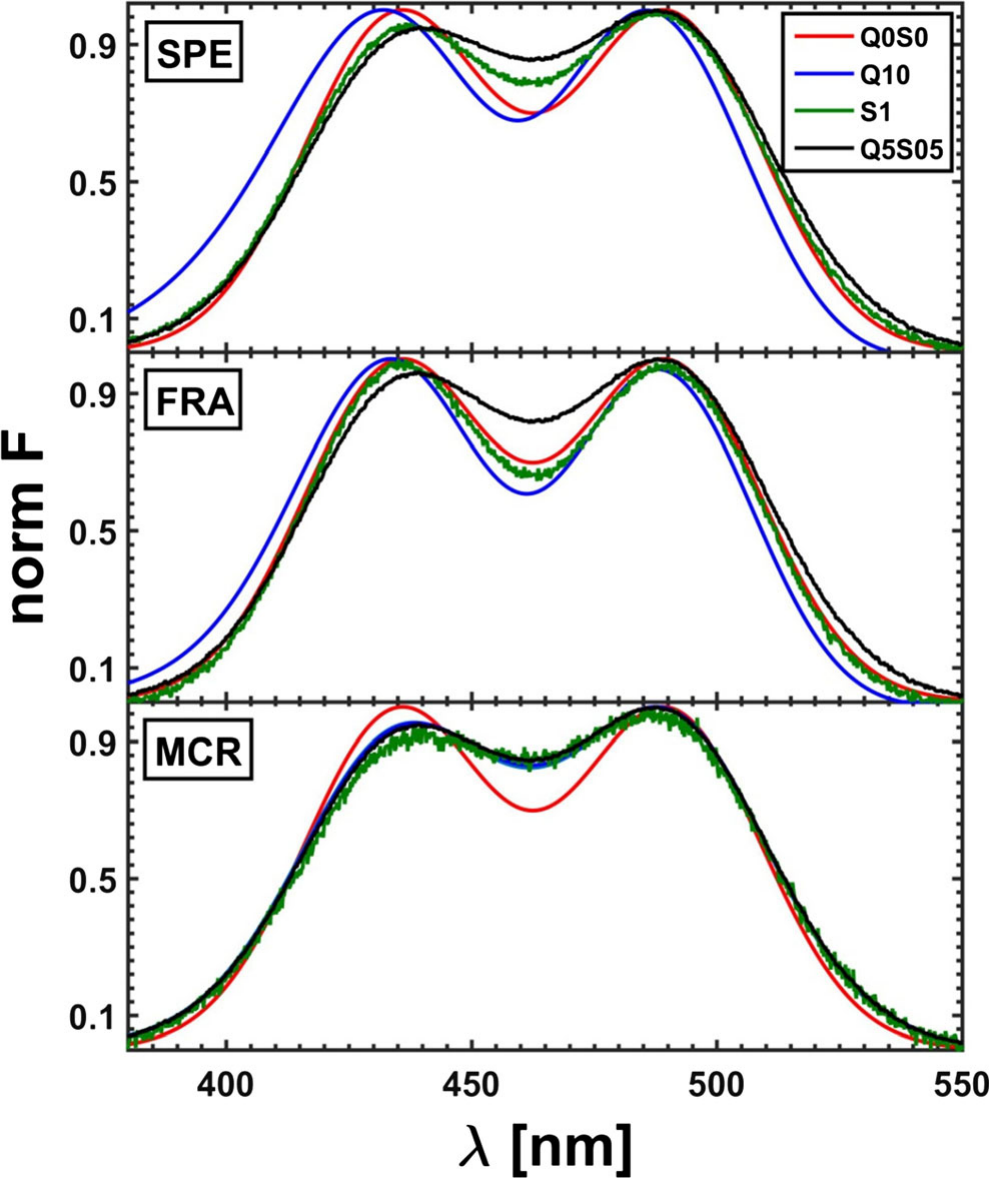
Effect of spectral (S ‰) and quencher concentration (Q %) noise on the shape of resolved spectrum of component C. The applied methods as in Fig. 18

The effect of the noise type and level on the shape of the resolved spectrum is demonstrated here on the example of the worst resolved spectrum of substance C (see Fig. 19).

**Fig. 20:**
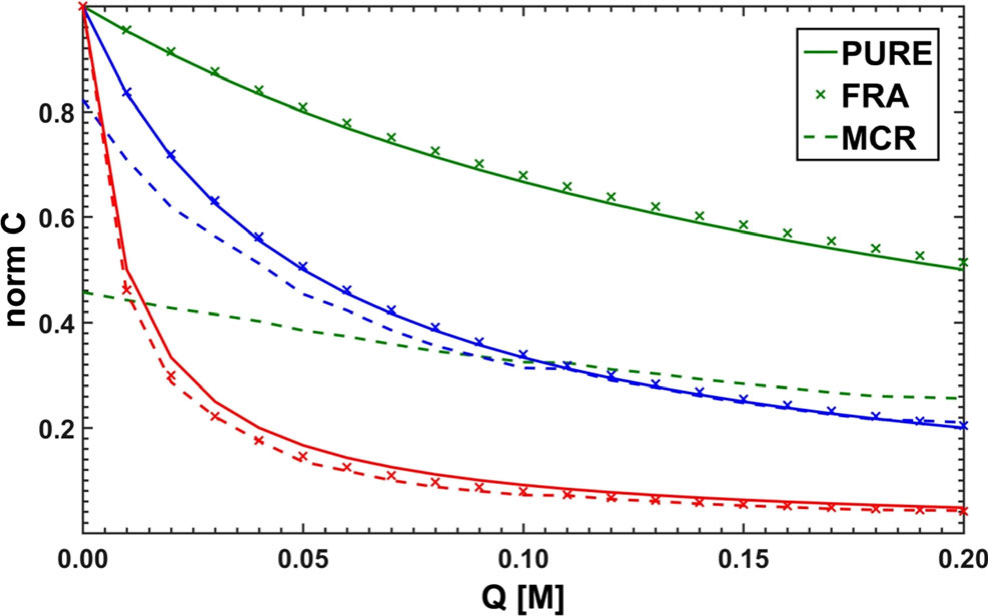
Optimized and normalized to maximum intensity decays of pure components. The applied methods as in Fig. 18

In addition, in Fig. 20 the obtained concentration profiles (or properly Stern-Volmer intensity decays) of pure fluorophores are shown revealing that a major difference can be noticed between the functional curves provided by the hard ‘fractional’ method and those digitalized curves retrieved using the hard-soft method of MCR-ALS in which only a non-negativity constant was imposed.

### Empirical Data

**Fig. 21:**
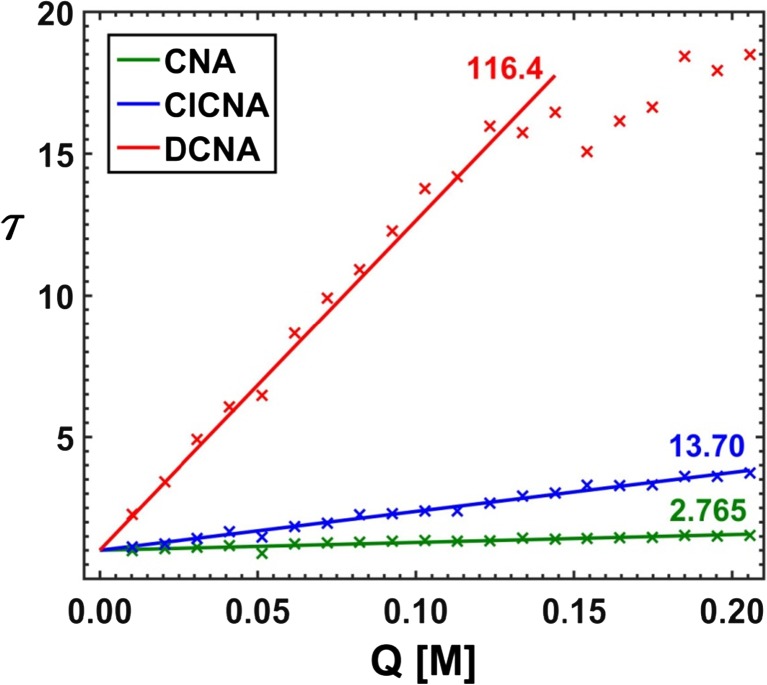
Stern-Volmer plots based on the results of *τ*-RAFA applied to empirical dataset without use of the SVD procedure

**Fig. 22:**
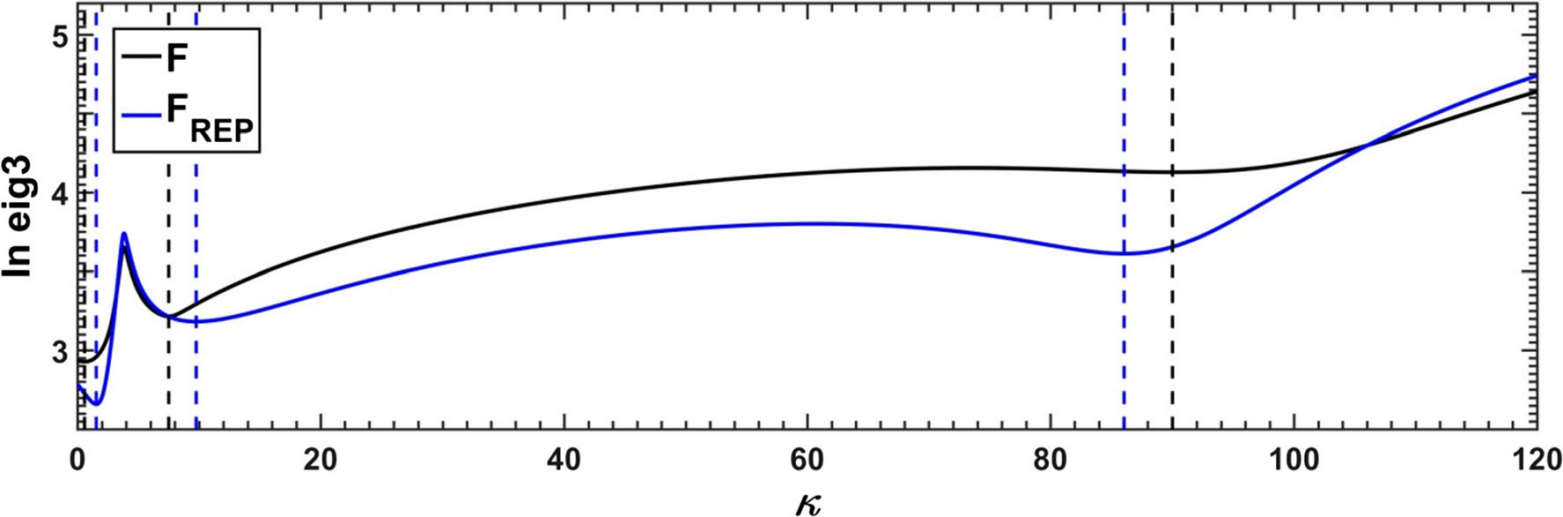
Stern-Volmer constants obtained using a ‘direct’ *κ*-RAFA applied to real dataset, not reproduced (F) and reproduced (F_REP_) with use of SVD procedure; excitation line at *λ* = 374 nm

As a final test, an attempt was made to resolve the empirical spectra (See “Experimental”). The whole process started with estimation of the Stern-Volmer quenching constants. First, the *τ*-RAFA method was applied, iterative parameter versus quencher concentration plots were drawn and the straight lines were fitted (Fig. 21). To allow a comparison, the results of the *κ*-RAFA approach applied to the data with and without reduced dimensionality were also taken into account (Fig. 22). Because the results of ‘indirect’ *τ*-RAFA method seemed to be more reliable (see previous section), only these were considered as appropriate for further processing.

**Fig. 23:**
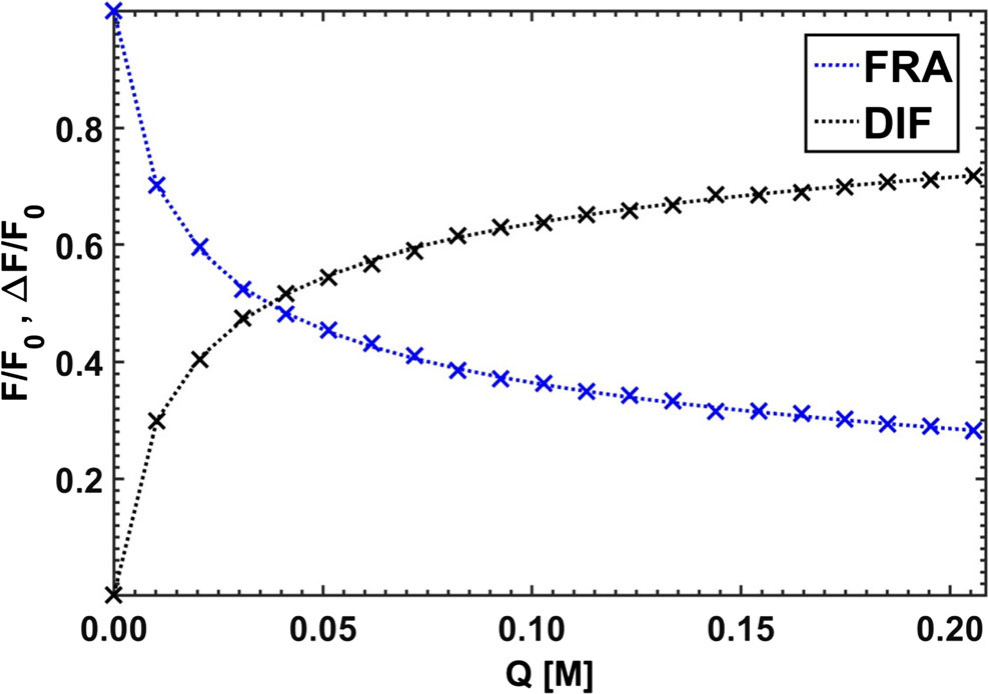
Curve fitting by ‘point’ type optimizations of real dataset (x) representing a fluorescence intensity decay at emission wavelength *λ* = 445 nm; FRA - Acuña method, and DIF – Lehrer method

In the next part of this study, which may also be called an overview of historical curiosities related to the estimation of the Stern-Volmer constants by means of ‘point’ methods, the best possible sets of coefficients for the Lehrer and Acuña schemes were determined using the nglm algorithm for two hundred different wavelengths at which the decays of the quenched fluorescence intensity were observed (Fig. 23). The averaged values of the Stern-Volmer constants obtained by the ‘point’ techniques are collected in Table 3 and compared with those determined for pure components and extracted using both versions of RAFA.

**Table 3:** Stern-Volmer constants obtained for real data using different methods

Method	K_CNA_	K_ClCNA_	K_DCNA_
EMP	2.044	13.85	122.7
*τ*-RAFA	2.765	13.70	116.4
*κ*-RAFA	0.580	7.50	90.4
FRA	0.901	9.03	113.8
DIF	0.438	15.98	127.8

^a^S-V constants in M

^b^EMP – determined for pure components, RAFA – obtained using two versions of RAFA, FRA/DIF – estimated with use of white ‘point’ methods

**Fig. 24:**
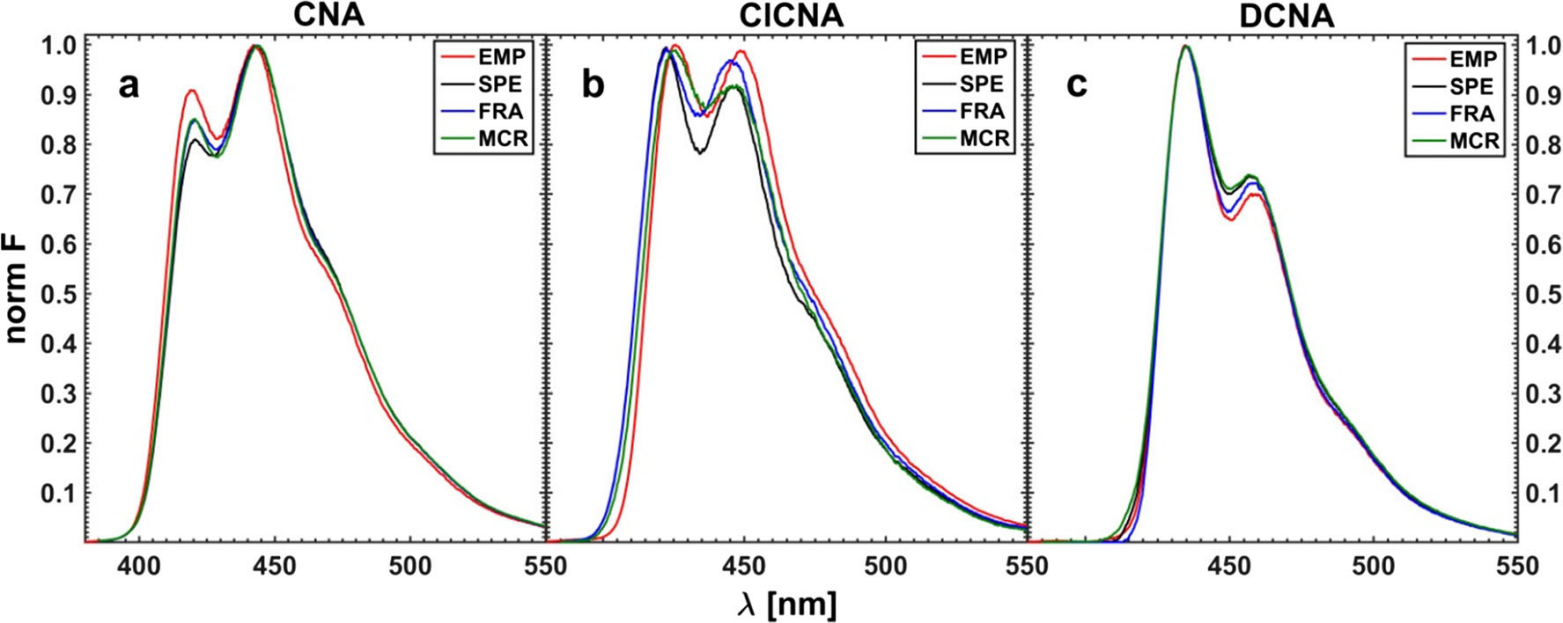
Averaged fluorescence spectra of all three components of a real system resolved from the mixture spectra with no use of the SVD procedure; EMP – measured spectrum of a pure component; SPE – spectrum obtained by means of ‘classical’ white method, FRA – yielded using fractional data, and MCR – produced by grey MCR-ALS method of Tauler et al. [42]

The last section of this study includes the resolution of three-component system of fluorescence quenching by means of white and grey methods of data modeling. The initial concentration matrix was constructed on the basis of the *τ*-RAFA factor analysis. At first, the raw data, not ‘smoothed’ by the SVD procedure, were analyzed. Prior to using the ‘fractional’ hard algorithm, the data range was reduced to 420–500 nm. The resolved emission profiles of pure components obtained for five different excitation lines by all the applied methods were then averaged since the shape of the fluorescence profile of each fluorophore, unlike the intensity, should remain unchanged regardless the applied excitation line. The resulting spectra of pure components are portrayed in Fig. 24.

The measured three-component spectra of quenched fluorescence upon preliminary SVD pretreatment were resolved as well. However, a difference between the results of both approaches (with and without the SVD data preprocessing) in the case of white methods was practically unnoticeable. Moreover, as regards the grey MRC-ALS algorithm, the emission profiles of single constituents resolved upon the preliminary use of the SVD procedure are even more divergent from the expected spectra (measured for individual components) than those obtained for raw experimental data of the fluorophore mixture.

**Fig. 25:**
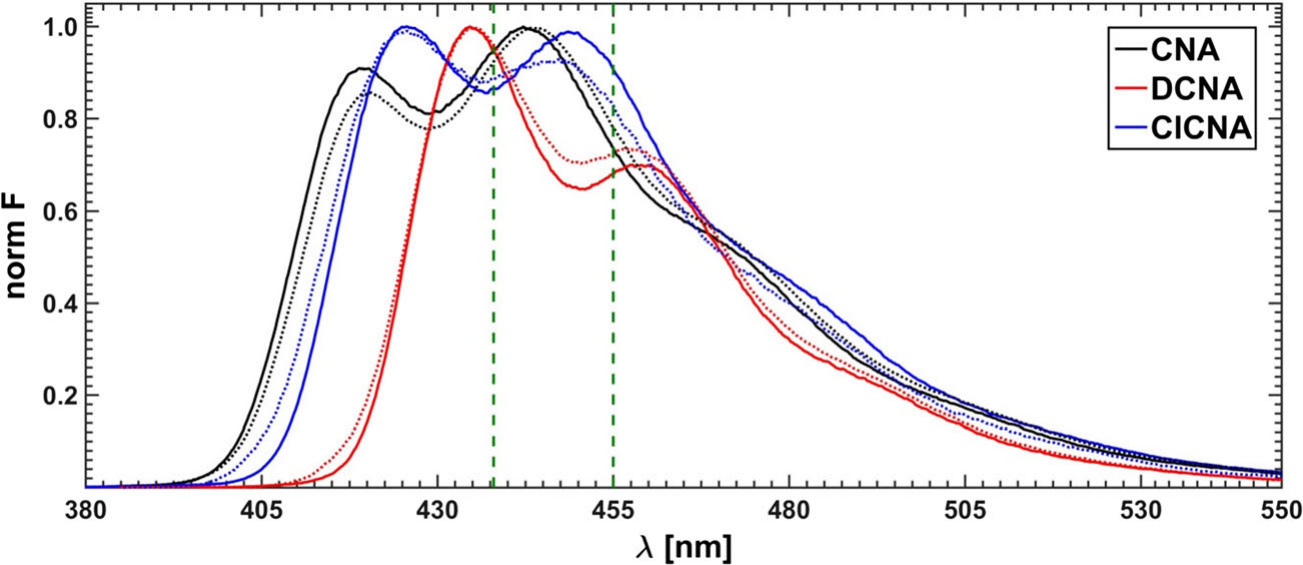
Comparison of measured (solid lines) and resolved (dashed lines) spectra of pure components. The latter are resolved with the use of Stern-Volmer constants determined from the quenched fluorescence measurements performed for single fluorophores

In order to assess, for assumed quencher concentrations, how inaccurate the whole procedure of sample preparation might be the recorded spectra of the fluorophore mixture have also been resolved on the basis of the concentration matrix generated for the Stern-Volmer constants determined by the analysis of fluorescence quenching of one component systems. The graphical outcome is depicted in Fig. 25.

## Conclusions

### Application of RAFA

The analysis of the obtained results reveals that the method of ‘indirect’ rank annihilation factor analysis, *τ*-RAFA, can be successfully employed to determine the number of components in the multi-component system of quenched fluorescence and to estimate their Stern-Volmer quenching constants. Imperfection in determining the quencher concentration seems to have rather negligible effect on the final outcome, while a spectral noise influence cannot be ignored and appears to be a main limitation of the method – the results of RAFA for the data with spectral noise level higher than 1‰, should not be considered as reliable.

The ‘direct’ *κ*-RAFA method is very ‘noise-prone’ – both concentration inaccuracy and spectral noise insert influence on the final results, which are, moreover, diverging from the expected ones. The *κ*-RAFA algorithm should only be used as a tool in pre-analysis of the collected data or to confirm findings already unveiled by other methods - the Authors suggest using both RAFA approaches concomitantly, after initial determination of the number of principal components.

### ‘Point’ Optimization Methods

The ‘point’ methods accounted for as rather historical episodes are yet still used even today, especially to pinpoint some specific fragments of proteins in biochemical systems as molecular markers of cancer or virus spread [45, 46]. Application of these methods allows for minimization of computational resources since it does not require a continuous recording of the fluorescence spectra. A basic knowledge of the measured system is, however, essential due to a high sensitivity of the optimization algorithm to initial values of the optimized parameters. The experimenter should at least be aware of what are the possible values of the Stern-Volmer quenching constants of individual fluorophores and which emission lines are, in a broad sense, the most suitable for the analysis – ‘point’ approaches are highly sensitive to the data noise level.

### White and Grey Methods of Data Modeling

It turns out that white algorithms of data modeling allow to resolve the composite fluorescence spectra with acceptable resemblance to the original pure component spectra. Shape of the calculated emission profile depends mainly on the spectral data noise level – calculations above 1‰ may be treated as uncertain. Concentration inadequacy, by contrast, results in shifting of the whole spectrum to lower or higher wavelengths. Fortunately, the initial values of the variable parameters entered to the optimization nglm algorithm do not have apparent influence on final outcome.

In order to evade, at least some of the mentioned above limitations, the range of the scrutinized noisy spectra should be reduced – this may easily be done through data transformation which is a part of the Acuña et al. matrix approach. Due to low costs of calculations and significant advantages, it is recommended to estimate an effective spectral range with the use of the ‘fractional’ data type technique prior to application of any hard optimization algorithms.

The grey MCR ALS approach operating on digitalized curves, i.e. curves with unknown functional forms, becomes independent of the assumed values of the quencher concentration which is an undeniable advantage of the method. Furthermore, the data noise at least up to the noise level of 1‰ seems to have a negligible effect on the final results, but the algorithm due to a decreased number of applied constraints is sensitive to initial entries of the concentration matrix – a pre-factor analysis should be performed. Despite the fact that the fluorescence spectra of single components resolved in this study by the MCR ALS approach are not so accurate as those obtained by hard modeling, the method remains undoubtedly a very useful tool for immediate evaluation of feasible solutions.

### General Conclusions

Both white and grey methods of data modeling combined with two RAFA approaches made it possible to resolve a system of three-component spectra of fluorescence quenching. The retrieved emission profile appeared to be the most accurate for the substance with the highest Stern-Volmer quenching constant while the worst reproduced spectrum is that referring to the fluorophore with the Stern-Volmer constant in between the two extreme values. This probably might be justified by imperfection of sample preparation but in the case of simulated system such explanation fails – presumably neither hard nor hard-soft algorithms are good enough to correctly resolve the complex fluorescence spectra in the region of strongly overlapping emission bands. Another, more credible, explanation is that the values of both low Stern-Volmer constants are too close to each other so this substantially hinders the process of yielding the properly resolved spectra through optimization. The obtained spectra are, however, in Authors’ opinion, sufficiently consistent with the reference synthetic or experimental spectra of pure components.
